# The SPA-cube framework: An integrated approach for analysing power dynamics in environmental governance

**DOI:** 10.1016/j.mex.2026.103814

**Published:** 2026-02-03

**Authors:** Muhammad Alif K. Sahide, Siswandi Siswandi, Nurhady Sirimorok, Micah R. Fisher, Grace Yee Wong, Maria Brockhaus

**Affiliations:** a Forest and Society Research Group (FSRG) of Faculty of Forestry, Universitas Hasanuddin, Makassar, Indonesia; b Matsunaga Institute for Peace and Conflict Resolution, School of Communication and Information, University of Hawai‘i, Honolulu, United States; c Research Institute for Humanity and Nature (RIHN), Japan; d International Forest Policy, University of Helsinki, Finland

**Keywords:** Power analysis, Environmental governance, Gaventa's power cube, Governmentality, Sequential power analysis

## Abstract

This article presents the SPA-Cube Framework, an integrated methodological protocol that addresses key limitations in power analysis for environmental governance. Traditional approaches often employ singular or static lenses, failing to capture power’s multidimensional, dynamic, and governmental nature. Our framework systematically integrates three complementary pillars: Gaventa’s Power Cube (typologizing visible, hidden, and invisible power), governmentality theory (revealing rationalities and technologies of rule), and Sequential Power Analysis (providing temporal sequencing and actor-centric focus). This synthesis enables researchers to simultaneously examine *when* and *how* power shifts, *what* forms it takes, and *why* it operates as it does across policy interventions. The framework helps explain paradoxical outcomes, such as how empowerment strategies can inadvertently reinforce existing structures. Developed and validated through a 36-month ethnographic study in Indonesia, it offers a novel tool for navigating complex power dynamics by providing a structured protocol that integrates temporal, typological, and governmental analyses into one workflow.

• Offers a replicable, step-by-step procedure for conducting integrated power analysis across policy cycles.
• Enables dynamic tracking of power across three phases: background conditions, intervention delivery, and post-intervention adjustments.
• Analyses visible, hidden, and invisible power with a focus on how technologies of rule shape subject formation.

## Graphical abstract

**Figure:**
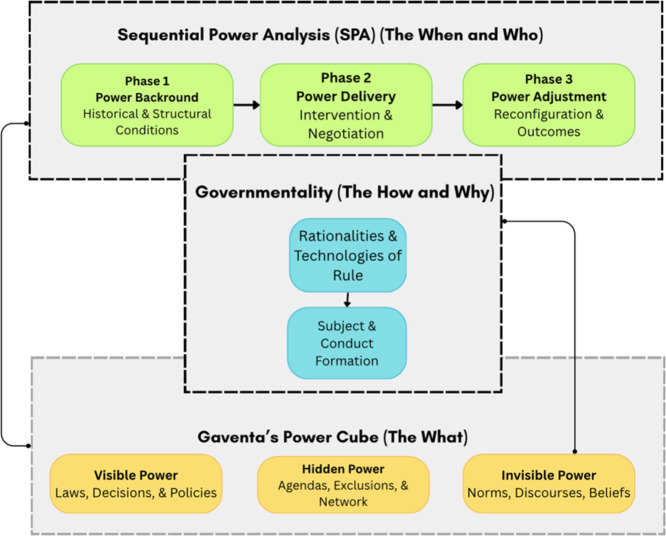

**Specifications table**

**Table:** 

**Subject area**	**Environmental Science**
**More specific subject area**	**Human ecology**
**Name of your method**	**SPA-Cube**
**Name and reference of original method**	**SPA-Cube is an original methodological framework that integrates three existing approaches: Gaventa's Power Cube** [12], **Governmentality theory** [13,14], **and Sequential Power Analysis** [11].
**Resource availability**	**-**

## Background

Power analysis in environmental governance confronts a persistent methodological challenge: the inherent complexity of capturing power's multidimensional and dynamic nature [1,2]. Traditional methodological approaches have typically privileged singular theoretical traditions, each illuminating certain facets of power while obscuring others [3,4]. Structural analyses effectively reveal institutional constraints but often neglect human agency and strategic action. Discursive approaches excel at uncovering ideological formations yet frequently miss material dimensions and resource distributions. Static frameworks, while methodologically convenient, fundamentally misrepresent power as a fixed attribute rather than a relational process that evolves through time and across contexts [5].

This methodological limitation becomes particularly acute in studying environmental interventions and policy implementations, where power relations undergo continuous transformation across different phases, scales, and dimensions [6]. Conservation programs, climate adaptation initiatives, and resource governance reforms often generate paradoxical outcomes where empowerment strategies inadvertently reinforce existing power asymmetries, or participatory approaches reproduce exclusionary practices [7]. Recent studies on social forestry in Indonesia, for instance, reveal how NGOs navigating power relations with state companies may shift strategies and motivations, sometimes aligning with powerful actors rather than local communities, thereby sustaining existing inequities [8]. Similarly, a recent application of a Sequential Power Analysis-Exclusion framework to social forestry in South Sulawesi revealed how participatory policies can be hijacked to enable corporate control through processes of ‘exclusion by inclusion’ across temporal phases, further underscoring the need for dynamic, integrated analysis [9]. Understanding these dynamics requires methodological approaches capable of tracking power’s temporal evolution while simultaneously analyzing its multiple manifestations and underlying rationalities.

The SPA-Cube Framework emerges as a methodological response to these challenges, integrating three complementary theoretical traditions into a coherent analytical protocol. It builds directly upon the foundation of Sequential Power Analysis (SPA), a framework developed to dissect the temporal stages of power background, delivery, and adjustment that shape outcomes in community forestry partnerships [10]. Sequential Power Analysis [11] provides the temporal scaffolding (the When and Who) to systematically track power dynamics across intervention phases. Gaventa’s Power Cube [12] offers a comprehensive typology (the What) to categorize different forms and expressions of power. Governmentality theory [13,14] reveals the underlying rationalities and technologies (the How and Why) that render power operative and effective. This integration enables researchers to examine how power transforms across temporal sequences, manifests in different forms, and operates through specific governmental technologies. This structured integration differentiates the SPA-Cube from hybrid approaches that merely list multiple theories. It provides a replicable analytical procedure that explicitly guides researchers in how to combine these lenses at each step of their investigation.

The framework’s development responds to several methodological gaps identified in environmental governance research. First, it addresses the temporal blind spot in power analysis by providing structured protocols for tracking power dynamics across policy cycles and intervention phases. Second, it overcomes the typological limitations of single-dimension approaches by systematically analyzing visible, hidden, and invisible power manifestations. Third, it bridges the theory-practice divide by offering operational tools for analyzing how governmental rationalities shape environmental subjects and governance outcomes. The framework’s design enables application across diverse environmental governance contexts, including climate policy implementation, natural resource management conflicts, conservation program evaluation, and sustainability transition governance. The framework is also highly applicable for dissecting the power dynamics within environmental discourses, such as the competing narratives among promoters, moderators, and opponents of REDD+ projects [15].

## Method details

### Theoretical framework and integration logic

**Fig. 1:**
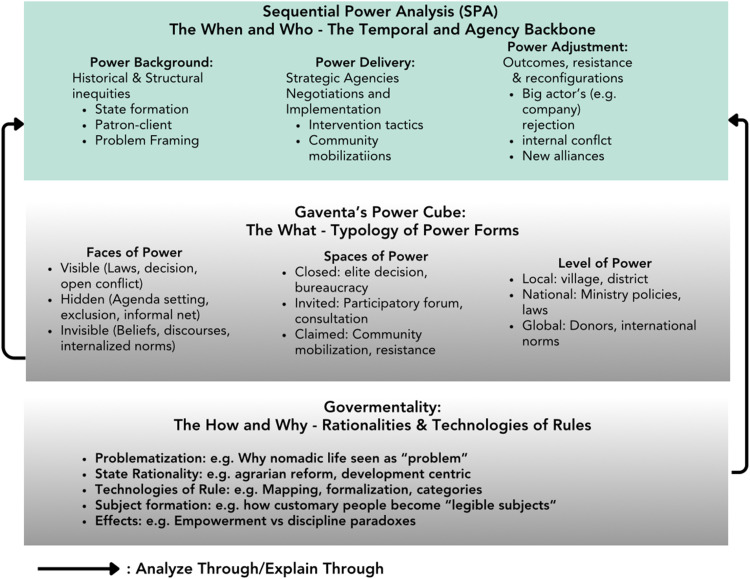
SPA-cube analytical framework: integrating temporal, typological and governmentality dimensions.

The SPA-Cube Framework's integration logic addresses a fundamental methodological challenge: how to systematically analyze power across its temporal, typological, and governmental dimensions without reducing complexity or sacrificing analytical precision. As illustrated in Fig. 1, the framework creates an integrated analytical space where these dimensions interact dynamically.

Fig. 1 visually represents the integrated framework showing three interconnected dimensions: (i) Sequential Power Analysis (*When & Who*) with its three-phase temporal structure; (ii) Power Cube (What) with its multidimensional typology of faces, spaces, and levels; and (iii) Governmentality (How & Why) with its focus on rationalities, technologies, and subject formation. Arrows indicate dynamic interactions and feedback loops between all dimensions.)*

### The when and who: Sequential power analysis operationalization

SPA [11] provides the temporal architecture for power analysis through three systematically linked phases: Power Background Analysis, Power Delivery Investigation, and Power Adjustment Assessment. Each phase requires specific analytical focus and methodological approaches.

**Power Background Analysis** requires investigators to reconstruct the historical and structural conditions preceding intervention. This involves four core tasks: (1) conducting historical reconstruction of institutional arrangements and structural inequalities, (2) mapping latent conflicts, historical grievances, and established patron-client networks through archival research and oral history interviews, (3) identifying path dependencies and institutional legacies that constrain contemporary agency, and (4) documenting initial resource distributions and established authority patterns.

**Power Delivery Investigation** focuses on intervention implementation through four methodological approaches: (1) real-time tracking of negotiation processes and strategy adaptations using participatory observation, (2) systematic documentation of meeting dynamics, decision-making processes, and agenda control mechanisms, (3) actor-network mapping to identify key intermediaries, brokers, and excluded parties, and (4) resource flow analysis to trace how material and symbolic resources are mobilized and deployed.

**Power Adjustment Assessment** examines post-intervention reconfigurations through four analytical strategies: (1) longitudinal tracking of emerging alliances, resistance strategies, and adaptation responses, (2) conflict transformation analysis to identify how old conflicts mutate and new conflicts emerge, (3) institutional ethnography of new governance arrangements and power reconfigurations, and (4) outcome distribution analysis to identify winners, losers, and unintended consequences.

### The what: Gaventa's power cube application

The Power Cube's multidimensional typology [12] requires systematic application across all SPA phases through three interconnected analytical dimensions: Faces of Power, Spaces of Power, and Levels of Power.

**Faces of Power Analysis** involves investigating power across three manifestations. This comprises: (1) **Visible Power Documentation** through systematically cataloging laws, policies, formal decisions, and regulatory frameworks via document analysis and legal review; (2) **Hidden Power Mapping** by identifying agenda control mechanisms, strategic exclusions, and informal networks through process tracing and network analysis; and (3) **Invisible Power Investigation** that uncovers dominant discourses, internalized beliefs, and ideological formations through discourse analysis and ethnographic immersion.

**Spaces of Power Examination** requires analyzing where power is exercised across three types of arenas. This includes: (1) **Closed Spaces Identification** that maps elite decision-making arenas, bureaucratic procedures, and technical committees through institutional analysis; (2) **Invited Spaces Documentation** that tracks stakeholder consultations, participatory processes, and multi-stakeholder platforms through process observation; and (3) **Claimed/Created Spaces Recognition** that identifies grassroots mobilizations, autonomous organizing, and counter-public spheres through movement analysis.

**Levels of Power Integration** necessitates examining power operations across different governance scales through three approaches: (1) **Cross-scalar Analysis** to track how decisions at global and national levels affect local implementation and vice versa; (2) **Vertical Integration Examination** to analyze how power operates across local, national, and global governance scales; and (3) **Scale-jumping Strategies Identification** to document how actors mobilize resources and alliances across different levels

### The how & why: Governmentality analysis

Governmentality [13,14] investigation focuses on the rationalities and technologies that make power operative, examining how governance shapes both institutions and subjectivities through three interconnected analytical components.

**Governmental Rationalities Analysis** involves unpacking the thought systems that justify governance interventions. This comprises: (1) **Problematization Identification** to analyze how certain behaviors, populations, or practices become framed as "problems" requiring intervention; (2) **Truth Regime Examination** to identify dominant knowledge systems, expert discourses, and evidence regimes that authorize certain interventions; and (3) **Calculative Rationality Documentation** to trace how complex social-ecological systems are rendered calculable and manageable.

**Governmental Technologies Investigation** requires examining the instruments through which governance is enacted. This includes: (1) **Technical Device Analysis** of how tools like participatory mapping, certification systems, or reporting mechanisms discipline conduct; (2) **Administrative Procedure Documentation** of how bureaucratic procedures, reporting requirements, and audit systems shape behavior; and (3) **Knowledge System Examination** of how monitoring systems, indicators, and evaluation frameworks produce particular truths.

**Subject Formation Tracking** involves analyzing how interventions reshape individual and collective subjectivities through: (1) **Identity Transformation Analysis** to document how interventions reshape community identities, social roles, and self-understandings; (2) **Conduct Regulation Examination** to track how new norms, expectations, and behavioral standards are institutionalized; and (3) **Agency Reshaping Investigation** to analyze how new forms of agency are enabled while others are constrained. For example, in a community based conservation program, Subject Formation Tracking might examine how villagers internalize a new identity as “conservation stewards”. This can be traced through their adoption of technical vocabularies, changed interactions with officials, and negotiation of this role within local hierarchies. In contrast, the framework is equally crucial for analyzing how historically loaded labels, such as “customary people”, a term with colonial origins often used to avoid recognizing Indigenous sovereignty, are reproduced or contested. Tracking this reveals how power operates not just by imposing new roles, but also by reinforcing or reshaping existing categories of identity and difference.

### Integrated analytical procedure: A step-by-step protocol

**Phase 1: Comprehensive Temporal Mapping (The When & Who)** involves: (1) constructing multi-layered timelines documenting intervention phases, key decisions, and critical junctures; (2) developing actor-network maps tracking organizational relationships, individual positioning, and alliance formations across time; (3) identifying turning points and moments of contingency where power relations were renegotiated or reinforced; and (4) documenting the rhythm and tempo of intervention processes across different temporal scales.

**Phase 2: Systematic Multidimensional Power Analysis (The What)** requires for each SPA phase conducting simultaneous investigation of: (1) visible power manifestations through systematic document analysis and decision process tracking; (2) hidden power operations through network analysis, agenda-setting examination, and exclusion identification; and (3) invisible power effects through discourse analysis, ideological critique, and ethnographic observation of internalization processes.

**Phase 3: Deep Governmentality Examination (The How & Why)** entails: (1) identifying specific problematizations that justified intervention and tracing their discursive construction; (2) analyzing how governmental technologies transformed complex realities into administrable problems; and (3) tracking subjectification processes through which actors internalized new identities and conduct norms.

**Phase 4: Integrative Cross-dimensional Synthesis** completes the protocol by: (1) examining interconnections between temporal sequences, power forms, and governmental rationalities; (2) identifying paradoxical outcomes and unintended consequences across different dimensions; (3) tracing reinforcement mechanisms where empowerment strategies reproduced existing power logics; and (4) developing integrated explanations for why interventions produced specific patterns of inclusion and exclusion.

### Practical implementation guidelines and analytical framework

#### Data collection strategy

The framework requires methodological triangulation combining four complementary approaches: (1) **Document Analysis** through systematic review of policy documents, meeting minutes, reports, and archival materials; (2) **Ethnographic Engagement** through long-term participatory observation, informal conversations, and situated learning; (3) **Interview Protocols** including semi-structured interviews, focus group discussions, and elite interviews; and (4) **Process Documentation** through real-time tracking of meetings, negotiations, and decision-making processes.

#### Analytical framework implementation

Table 1 (SPA-Cube Analytical Framework: Operationalizing Integrated Power Analysis) provides the complete analytical framework connecting SPA phases with Power Cube dimensions and Governmentality concepts, with specific guiding questions for each analytical dimension.

**Table 1:** SPA-cube analytical framework: Operationalizing integrated power analysis.

Analytical Dimension	Guiding Questions	Power Cube Dimensions (The WHAT)	Governmentality Concepts (The HOW & WHY)
**POWER BACKGROUND (The WHEN)**	How are historical and structural inequalities formed and institutionalized? What state rationalities position certain lifestyles or practices as problematic? What historical technologies of governance established path dependencies?	**Faces**: Visible (laws, property regimes), Hidden (historical patronage, established networks), Invisible (dominant discourses, cultural norms) **Spaces**: Closed (colonial administration, elite circles), Invited (historical consultations, past reforms) **Levels**: Local (customary institutions), National (state formation), Global (colonial legacies)	Problematization of specific populations, Historical technologies of settlement and control, State rationalities of improvement and development, Path dependencies in governance arrangements
**POWER DELIVERY (The WHO)**	What strategies and tactics do different actors employ to navigate power structures? How do governmental technologies reshape subjectivities and social relations? What negotiation and adaptation processes characterize implementation?	**Faces**: Visible (formal negotiations, mapping exercises), Hidden (informal lobbying, strategic alliances), Invisible (identity reconstruction, discourse reframing) **Spaces**: Invited (participatory processes, multi-stakeholder platforms), Claimed (autonomous organizing, resistance spaces) **Levels**: Local (community mobilization), National (policy advocacy), Global (transnational networking)	Technologies of legibility (mapping, categorization), Subject formation (community representatives, environmental citizens), Governmentality of recognition (authenticity performance, cultural production), Calculative rationalities (cost-benefit analysis, indicator development)
**POWER ADJUSTMENT (The THEN)**	How do power relations reconfigure following intervention? What forms of resistance, adaptation, and counter-conduct emerge? Who benefits and who loses from new arrangements? What unintended consequences and paradoxical outcomes materialize?	**Faces**: Hidden (corporate resistance, bureaucratic inertia), Invisible (internalized conflicts, identity crises) **Spaces**: Closed (corporate boardrooms, ministry offices), Claimed (protest movements, alternative institutions) **Levels**: Local (village conflicts), National (policy feedback), Global (certification impacts)	Counter-conduct (everyday resistance, alternative practices), Unintended effects (perverse incentives, moral hazards), Subjectivity crises (identity conflicts, role confusion), Adaptive governance (learning processes, institutional innovation)

#### Iterative analytical process

Implementation follows four iterative steps: (1) developing an integrated coding framework that cross-references SPA phases with Power Cube dimensions; (2) conducting abductive analysis moving iteratively between empirical data and theoretical concepts; (3) employing comparative analysis across cases and contexts to identify patterns and variations; and (4) maintaining reflexive documentation of analytical decisions and interpretive frameworks.

#### Framework adaptation and practical application

The SPA-Cube Framework is designed to be modular and adaptable. While its full analytical rigor is demonstrated through long-term ethnography, researchers can strategically tailor its application depending on their study's scope, resources, and objectives. The following guidelines outline two primary adaptation pathways: adaptation for different research goals, and adaptation for resource-constrained settings.

1. Adaptation for Different Research Goals

The framework can be tailored for five distinct research applications while maintaining its core integrative logic:

  - **Short-term Policy Analysis**: Condense the temporal phases while maintaining multidimensional analysis. Focus intensively on the Power Delivery phase of a specific policy process, using Table 1 to ensure visibility across power types and governmentality.
  - **Comparative Case Studies**: Maintain consistent analytical dimensions (the columns of Table 1) across different cases to enable structured comparison of how power dynamics play out in varied contexts.
  - **Participatory Action Research**: Engage communities as co-analysts. Facilitate workshops where stakeholders collaboratively map power dynamics across the SPA-Cube dimensions, using the framework to inform strategy development.
  - **Program Evaluation**: Assess intervention effectiveness not just by stated goals, but by tracking shifts across multiple power dimensions (visible, hidden, invisible) and subject positions before and after implementation.
  - **Institutional Analysis**: Focus on organizational dynamics and governance arrangements by applying the framework to trace how institutional rationalities and technologies shape power relations within and between organizations.

2. Adaptation for Resource-Constrained Studies

For projects with limitations in time, funding, or data access, the framework can be applied through strategic scaling:

- **Temporal Condensation**: The three-phase sequence can be streamlined. Establish the Power Background through retrospective interviews and archival analysis rather than longitudinal observation. Concentrate primary empirical data collection on a critical Power Delivery period (e.g., a key negotiation). Assess Power Adjustment through forward-looking scenarios, expert elicitation, or shorter-term follow-ups.
- **Dimensional Prioritization**: When comprehensive data collection on all dimensions is impossible, identify and prioritize a pivotal analytical thread. For example, trace the interaction between one Power Cube dimension (e.g., Hidden Power in agenda-setting) and one Governmentality component (e.g., Problematization) across the condensed timeline. This yields deep, integrated insights on a specific power mechanism.
- **Collaborative and Secondary Data Methods**: Leverage participatory workshops to generate collective analysis, or conduct a systematic literature review/synthesis of existing case studies to populate the analytical matrix in Table 1.

*Guiding Adaptation Principle:* The essential principle is to preserve the framework's integrative intent. Even in scaled-down applications, researchers should ensure that questions from different columns of Table 1 (temporal phases, power forms, governmental rationalities) are in dialogue. The goal is to avoid reverting to a single one-dimensional analysis while making the protocol feasible.

## Method validation

The SPA-Cube Framework was developed, refined, and validated through its application in a 36-month critical ethnographic study examining the recognition process of the Punan Batu, a nomadic hunter gatherer community in Kalimantan, Indonesia. This empirical application substantiates the framework’s value by demonstrating its capacity to generate nuanced insights that conventional, single dimension power analyses would likely miss. [A detailed empirical analysis utilizing this framework, which forms the basis of this validation, is presented in a companion research article currently co-submitted to *Land Use Policy*]

### Validation through analytical output

The framework's structured procedure enabled the systematic uncovering of a core recognition paradox. While visible power analysis documented the success in securing formal legal recognition for the community, the integrated analysis of hidden and invisible power, sequenced through the Power Delivery and Adjustment phases, revealed how the very participatory technologies used for empowerment, such as mapping and institutional formalization, also functioned as governmental technologies. These technologies disciplined nomadic mobility, reterritorialized fluid spatial practices known as *adap*, and reshaped egalitarian social structures into state legible forms. This finding that empowerment and disciplinary governmentality are co constitutive validates the framework’s core integrative logic.

### Validation through explanatory power

The framework proved effective in tracing why interventions produced specific, often unintended, outcomes. For instance, by applying the Power Cube's typology across the SPA sequence, the analysis could trace how hidden power in historical patron client networks during the Power Background phase was not dissolved but reconfigured in the Power Adjustment phase into new forms of intermediary dependency post recognition. Similarly, the governmentality lens explained invisible power transformations, such as how state led problematization of nomadism was replaced by an NGO facilitated discourse of “last nomads”, creating new internal identity tensions within the community. The framework's tripartite integration provided a coherent explanation for these complex, multi layered outcomes.

### Validation through methodological utility

The application confirmed the practical utility of the framework's components. The Sequential Power Analysis scaffold ensured historical and temporal dynamics were not overlooked. The Power Cube typology provided a consistent coding matrix to categorize diverse data, from legal documents to interview transcripts, across visible, hidden, and invisible dimensions. The governmentality lens offered the critical depth to move beyond identifying what forms of power were present to understanding how and why they operated through specific rationalities, for example agrocentricity, and technologies, for example mapping. This structured yet flexible protocol allowed for a comprehensive analysis of a highly complex governance puzzle.

Therefore, the empirical application in a real-world, contentious environmental governance case provides robust validation. It demonstrates the framework’s ability to integrate disparate theoretical insights into a coherent analytical process, reveal paradoxical and non linear power dynamics, and offer a replicable protocol for researchers facing similar complexities in other contexts of environmental governance, policy implementation, and socio ecological struggle.

## Limitations

While comprehensive, the SPA-Cube Framework presents several methodological limitations that researchers should consider. First, its integrated nature demands significant theoretical sophistication, which may pose challenges for early-career researchers or those without interdisciplinary training. Second, comprehensive implementation requires extensive data collection across multiple dimensions, time periods, and governance levels, making it resource-intensive in terms of time, funding, and research capacity. Third, the framework's effectiveness may vary across different political contexts, governance arrangements, and intervention types, requiring careful contextual adaptation. Fourth, full application necessitates longitudinal engagement that may not be feasible in time-constrained projects. Finally, simultaneously tracking multiple power dimensions creates analytical challenges and interpretation complexities that require careful methodological management.

Consequently, the framework may be less suitable for rapid policy assessments with tight deadlines and limited data access, contexts with severe data limitations or research access constraints, studies exclusively focused on single power dimensions or theoretical traditions, and projects without adequate interdisciplinary expertise or supervisory capacity.

## Ethics statements

This research involved human subjects through ethnographic fieldwork, in-depth interviews, and participatory observation. All research procedures involving human participants were conducted in accordance with the ethical standards of the institutional review board and with the 1964 Helsinki Declaration and its later amendments. Prior to participation, informed consent was obtained verbally and/or in writing from all individual participants, including Punan Batu community members, government officials, NGO staff, and other key informants. Participants were informed of the study's purpose, the voluntary nature of their participation, their right to withdraw at any time, and the measures taken to ensure confidentiality and anonymization of their data. Special care was taken to ensure that consent was obtained in a culturally appropriate manner, using local languages and with the assistance of trusted interpreters where necessary.

## CRediT author statement

**Muhammad Alif K. Sahide:** Conceptualization, Methodology, Writing – original draft, Validation. **Siswandi Siswandi:** Conceptualization, Methodology, Validation. **Micah R. Fisher:** Conceptualization, Validation, Writing – review & editing. **Grace Yee Wong:** Supervision, Validation. **Maria Brockhaus:** Supervision, Validation.

## Declaration of competing interest

The authors declare that they have no known competing financial interests or personal relationships that could have appeared to influence the work reported in this paper.

## Data Availability

The data that has been used is confidential.

## Appendix Supplementary materials

Image, application 1

## References

[1] Shackleton R.T., Walters G., Bluwstein J., Djoudi H., Fritz L., Lafaye de Micheaux F., Kull C.A. (2023). Navigating power in conservation. Conserv. Sci. Pract..

[2] Maryudi A., Sahide M.A. (2017). Research trend: power analyses in polycentric and multi-level forest governance. For. Policy Econ..

[3] Hall D., Hirsch P., Li T.M. (2011).

[4] Li T.M. (2007).

[5] Cleaver F. (2012).

[6] Fisher M.R., van der Muur W. (2020). Misleading icons of communal lands in Indonesia. Asia Pac. J. Anthropol..

[7] Lund C., Boone C. (2013). Introduction: land politics in africa — constituting authority over territory, property and persons. Afr.: J. Int. Afr. Inst..

[8] Ragandhi A., Hadna A.H., Setiadi S., Maryudi A. (2025). Nongovernmental organizations in the design and implementation of social forestry programs in indonesia: interests, power and strategies. For. Soc..

[9] Saputra I., Faturachmat F., Muin A.V.F., Sirimorok N., Sahide M.A.K. (2025). Sequencing the political forest: power, exclusion, and the corporate hijacking of social forestry in Indonesia. For. Policy Econ..

[10] Sahide M.A.K., Fisher M.R., Supratman S., Yusran Y., Pratama A.A., Maryudi A., Kim Y.S. (2020). Prophets and profits in Indonesia's social forestry partnership schemes: introducing a sequential power analysis. For. Policy Econ..

[11] Sahide M.A.K., Fisher M.R., Verheijen B., Maryudi A., Kim Y.S., Wong G.Y. (2020). Sequential power analysis framework in assessing social forestry outcomes. MethodsX.

[12] Gaventa J. (2006). Finding the spaces for change: a power analysis. IDS Bull..

[13] Foucault M., Burchell G., Gordon C., Miller P. (1991). The foucault effect: studies in governmentality.

[14] Agrawal A. (2005).

[15] Chorito A.B., Assefa E. (2025). Voices in the forest: unraveling REDD+ discourse and narratives in the bale Eco-region, Ethiopia. For. Soc..

