# Supplementary material for: The SPA-cube framework: An integrated approach for analysing power dynamics in environmental governance
Source: MethodsX. 2026 Feb 3;16:103814. doi: 10.1016/j.mex.2026.103814 (PMC12906020; doi:10.1016/j.mex.2026.103814)
Supplement: Supplementary file 1 [file mmc1.pdf]

Land Use Policy

Navigating Power: The Role of a Conservation NGO in Recognising and Securing Customary Forest Rights for Borneo's Last Nomads

--Manuscript Draft--

**Manuscript Number:**	LUP-D-25-03824
**Article Type:**	Full Length Article
**Keywords:**	Agrocentric Governmentality; Customary Forest Recognition; Sequential Power Analysis (SPA); Non-agrarian Nomads; Power Navigation
**Corresponding Author:**	Muhammad Alif K. Sahide<br>Universitas Hasanuddin Fakultas Kehutanan<br>Indonesia
**First Author:**	Siswandi Siswandi
**Order of Authors:**	Siswandi Siswandi
	Nurhady Sirimorok
	Micah R. Fisher
	Grace Yee Wong
	Maria Brockhaus
	Muhammad Alif K. Sahide
**Abstract:**	The legal recognition of Customary Forest (Hutan Adat) in Indonesia, although presented as a progressive reform policy, centers around a state-centric understanding of community land tenure. It is agrocentric in its paradigm, envisioning tools to support sedentary-agrarian communities. This research employs a critical ethnographic approach to analyse the struggles of a very different community in obtaining legal recognition, namely the last nomadic groups of Kalimantan, the Punan Batu.<br>Using an integrated SPA-Cube Framework, this study examines the ways that agrocentric governmentality works to marginalise the Punan, albeit gaining formal state recognition. Indeed, we chart the ways Punan nomadic ontologies are rendered illegible. The analysis begins by examining the power background, revealing historical and structural inequalities rooted in state policies that systematically problematise nomadic ways of life. It then investigates the power delivery strategies of Yayasan Konservasi Alam Nusantara (YKAN) in their aims to leverage scientific research and participatory mapping. While these tactics succeeded in securing district-level support, they also served as government technologies to discipline the Punan Batu's mobilities by redefining them into state-recognisable subjects, re-territorialising their fluid spatial practices and formalising egalitarian social structures and practices. Finally, the analysis explores processes of power adjustment, revealing that interventions provoke retaliation from timber companies and agrarian settlers, entrenching patron-client networks to generate momentum for internal dissent. The first author'spositionality as a YKAN insider enabled rich, three-year-long participatory observation, providing deep access and unique insights. These insider perspectives were tempered through the collaborative research approach and prioritised an overallcritical reflexivity in engagement and analysis. Conceptually, this study contributes to environmental governance by demonstrating how the SPA-Cube Framework illuminates the paradoxes of recognition, where empowerment strategies can inadvertently reinforce the very governmental logics they challenge. Practically, it highlights the limitations of current recognition paradigms and offers critical insights for NGOs and policymakers.

Powered by Editorial Manager® and ProduXion Manager® from Aries Systems Corporation

To: The Editor in Chief
Land Use Policy

Subject: Submission of Manuscript Entitled "Navigating Power: The Role of a Conservation NGO in Recognising and Securing Customary Forest Rights for Borneo's Last Nomads"

Dear Editor,

We are pleased to submit our original research article, "**Navigating Power: The Role of a Conservation NGO in Recognising and Securing Customary Forest Rights for Borneo's Last Nomads**," for consideration for publication in *Land Use Policy*.

This study offers a critical, empirically grounded analysis of the complex power dynamics shaping the recognition of customary forest rights for non agrarian nomadic communities in Indonesia. Through a 36 month ethnography of the Punan Batu, one of Borneo's last active hunter gatherer societies, we examine how the well intentioned intervention of a conservation NGO, Yayasan Konservasi Alam Nusantara (YKAN), navigates state structures to secure legal recognition.

Our analysis reveals a profound recognition paradox. While participatory mapping and legal formalisation successfully delivered district level recognition, these same strategies also acted as governmental technologies that disciplined nomadic mobilities, re territorialised fluid spatial practices, and reshaped egalitarian social structures into state legible forms. The study further documents how recognition triggers new conflicts with timber companies, reinforces historical patron client networks, and generates internal dissent within the community.

Methodologically, the paper introduces and applies the SPA Cube Framework, an innovative integration of Sequential Power Analysis for temporal agency, Gaventa's Power Cube for typologies of visible, hidden, and invisible power, and Governmentality theory for rationalities and technologies of rule. This framework allows us to systematically trace how power operates, transforms, and produces paradoxical outcomes across the recognition process.

In line with advancing methodological transparency and utility, a detailed standalone methods article describing the SPA Cube Framework, including its analytical protocol, operational guidelines, and application toolkit, has been prepared as a companion piece. This methods paper is being co submitted concurrently to *MethodsX* (Elsevier), with the aim of supporting wider application of this integrated approach in environmental governance research.

We believe this manuscript strongly aligns with *Land Use Policy*'s focus on critical land governance, power relations, and the political ecology of resource recognition. It offers both theoretical contributions to understanding the politics of recognition and practical insights for NGOs, policymakers, and practitioners working at the intersection of indigenous rights, conservation, and sustainable land use.

This manuscript is not under consideration elsewhere, and all authors have approved its submission. We have no conflicts of interest to disclose.

Thank you for your time and consideration. We look forward to your response.

Sincerely,
Muhammad Alif K. Sahide (Corresponding Author/ On behalf of all co-authors)
Forest and Society Research Group, Universitas Hasanuddin
Email: muhammad.alif@unhas.ac.id

Highlights

- Reveals the paradox of state recognition for Indonesia's last nomadic hunter-gatherers.
- Uses the novel SPA-Cube Framework to analyze power dynamics in environmental governance.
- Shows how NGO-led empowerment strategies can inadvertently discipline nomadic communities.
- Demonstrates the persistence and reconfiguration of patronage despite legal recognition.
- Highlights the ontological clash between agrocentric state logic and nomadic ways of life.

Title page with author details

Navigating Power: The Role of a Conservation NGO in Recognising and Securing Customary Forest Rights for Borneo's Last Nomads

Siswandi Siswandi[1], Nurhady Sirimorok[1], Micah R. Fisher[2], Grace Yee Wong[3], Maria Brockhaus[4], Muhammad Alif K. Sahide[1]

[1] Forest and Society Research Group (FSRG) of Faculty of Forestry, Universitas Hasanuddin, Makassar, Indonesia
[2] Matsunaga Institute for Peace and Conflict Resolution, School of Communication and Information, University of Hawai‘i, Honolulu, United States
[3] Research Institute for Humanity and Nature (RIHN), Japan
[4] International Forest Policy, University of Helsinki, Finland

Manuscript without author details Click here to view linked References

Navigating Power: The Role of a Conservation NGO in Recognising and Securing Customary Forest Rights for Borneo's Last Nomads

Abstract.

The legal recognition of Customary Forest (Hutan Adat) in Indonesia, although presented as a progressive reform policy, centers around a state-centric understanding of community land tenure. It is agrocentric in its paradigm, envisioning tools to support sedentary-agrarian communities. This research employs a critical ethnographic approach to analyse the struggles of a very different community in obtaining legal recognition, namely the last nomadic groups of Kalimantan, the Punan Batu. Using an integrated SPA-Cube Framework, this study examines the ways that agrocentric governmentality works to marginalise the Punan, albeit gaining formal state recognition. Indeed, we chart the ways Punan nomadic ontologies are rendered illegible. The analysis begins by examining the **power background**, revealing historical and structural inequalities rooted in state policies that systematically problematise nomadic ways of life. It then investigates the **power delivery** strategies of Yayasan Konservasi Alam Nusantara (YKAN) in their aims to leverage scientific research and participatory mapping. While these tactics succeeded in securing district-level support, they also served as government technologies to discipline the Punan Batu's mobilities by redefining them into state-recognisable subjects, re-territorialising their fluid spatial practices and formalising egalitarian social structures and practices. Finally, the analysis explores processes of **power adjustment**, revealing that interventions provoke retaliation from timber companies and agrarian settlers, entrenching patron-client networks to generate momentum for internal dissent. The first author's positionality as a YKAN insider enabled rich, three-year-long participatory observation, providing deep access and unique insights. These insider perspectives were tempered through the collaborative research approach and prioritised an overall critical reflexivity in engagement and analysis. Conceptually, this study contributes to environmental governance by demonstrating how the SPA-Cube Framework illuminates the paradoxes of recognition, where empowerment strategies can inadvertently reinforce the very governmental logics they challenge. Practically, it highlights the limitations of current recognition paradigms and offers critical insights for NGOs and policymakers.

Keywords: Agrocentric Governmentality, Customary Forest Recognition, Sequential Power Analysis (SPA), Non-agrarian Nomads, Power Navigation

1. Introduction

The global struggle for indigenous land rights represents a fundamental clash of worlds. In recent years, counter-movements have gained traction in reclaiming land rights (Hall et al., 2011). In Indonesia, some indigenous communities have gained legal rights to recognition by engaging in agricultural development models. For the Punan Batu, the question of rights and livelihoods raises new conceptual and empirical questions. Despite constitutional assurances to indigenous communities in Indonesia, the state's operational logic prioritizes rights alongside its broader development models driven by commodity agriculture, using agrarian reforms as a tool to transform diverse landscapes into controlled plantation systems (Li, 2020; Scott, 1998).

This approach, which we call an "agrocentric framework", we initially hypothesized may systematically invalidate non-agrarian nomadic societies. For communities like the Punan Batu of Kalimantan, territory is not a bounded space to be owned and farmed, but a dynamic living space, or *adap,* sustained through seasonal mobility, hunting and gathering grounds, and spiritual sites.

We explored the extent to which their nomadic ontology might become rendered illegible to the state, as well as its implications. In particular, we were concerned about the way the state would see these longstanding practices as a problem to be solved rather than a way of life to be respected (Scott, 1998). The Punan Batu, one of Borneo's last active hunter-gatherer communities (Lansing et al., 2022; Kusuma et al., 2023), have gained significant support for legal recognition to their rights and territory, but we suggest that this may create a multi-front crisis. For broader, context, the land use changes unfolding across Kalimantan also threaten the existence of the Punan Batu by a relentless "territorial squeeze" from logging and palm oil concessions, land markets, agrarian settlers, and a deeply entrenched patron-client system with the heirs of the Bulungan Sultanate capitalising on legal vulnerabilities.

Therefore, this research argues that indigenous land rights is not merely about land conflict, but a deeper, more fundamental power struggle over recognition and legibility. The intervention by Yayasan Konservasi Alam Nusantara (YKAN) to facilitate the recognition of the Punan Batu as a *masyarakat hukum adat* (MHA, customary law community) and propose their Customary Forest (*Hutan Adat*), while well-intentioned, serves as a critical lens through which to examine narratives and outcomes of struggle. This study posits that recognition is not a neutral technical exercise but a contested political arena in which power is navigated, negotiated, and reconfigured.

To dissect these complex dynamics, we employ an integrated framework we term the **SPA Cube Framework**. This approach layers the typological insights of Gaventa's (2006) Power Cube, which distinguishes between visible, hidden, and invisible power, and the analytical depth of Governmentality (Foucault, 1991; Li, 2007), which reveals the underlying rationalities and technologies of rule onto the chronological and actor-centred scaffold of Sequential Power Analysis, or SPA (Sahide et al., 2020a,b). This synthesis allows us to move beyond a static analysis to ask not just *when* and *how* power shifts, but *who* drives these changes and *what* forms of power they wield. The Punan Batu case laid out in this paper presents an analytical challenge that reveals why conventional approaches to land rights are insufficient for addressing fundamental concerns for non-agrarian nomads. Specifically, this study asks: How do power relations influence the recognition process for a nomadic community? What strategies do actors, particularly YKAN, employ to navigate these power structures, and what agency do they exercise? Furthermore, what changes in the wake of recognition?

To address these questions we begin by elaborating our integrated SPA Cube Framework and our data collection approach applying qualitative and ethnographic methods. We then present our findings through three sequences of SPA: examining the power background of historical marginalisation, YKAN's power-delivery strategies and advocacy, and the subsequent power adjustments and paradoxes of recognition. The discussion explores the issues around our identification of what we call the agrocentric model influencing land rights recognition policy, the NGO's role as a power broker in enacting policy and practice, the persistence of patronage, and the unintended consequences of governmental technologies. We conclude by reflecting on the theoretical and practical implications for securing the rights of non-agrarian societies against in an agrocentric state polity.

2. An Integrated Framework: Layering the Power Cube and Governmentality on to Sequential Power Analysis

To comprehensively analyse the complex power dynamics in the struggle for the rights of the Punan Batu and their forest territories, this study proposes an integrated analytical framework that we term the SPA Cube Framework. This framework strategically merges three theoretical pillars to examine nuances of power relations in environmental governance. It layers the typological

insights from Gaventa's Power Cube and the mechanistic logic of Governmentality within the chronological structure of Sequential Power Analysis (SPA), enabling a multidimensional examination of when and how power shifts happen, what forms these powers take, and how and why these power relations operate and produce their effects. The visual representation in Figure 1 illustrates the integrated nature of this framework.

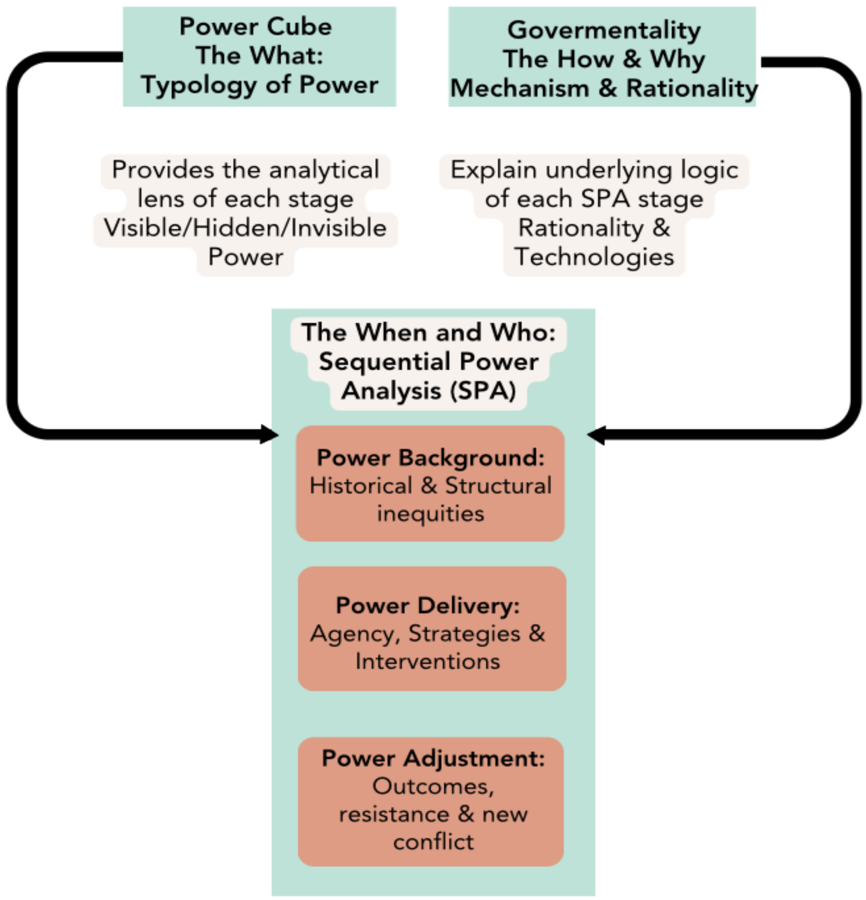

Figure 1. An Integrated Analytical Framework for SPA-Cube

While SPA provides the chronological framework, Gaventa's (2006) Power Cube offers the essential typology for categorising different forms of power. This perspective enables us to recognise not only that power is exercised but also how it functions across the SPA sequence. The Power Cube identifies three core dimensions: Visible Power (observable decision making, formal rules, and regulations), Hidden Power (controlling the political agenda and excluding issues from decision-making processes), and Invisible Power (shaping beliefs, perceptions, and preferences through ideologies and discourses). By applying this typology to each SPA stage, we can systematically determine whether power operates through open coercion, hidden agenda setting, or the subtle shaping of consciousness and identities.

2.1. The When and Who: Sequential Power Analysis as Temporal Scaffold and Agency

The foundation of this framework is **Sequential Power Analysis (SPA)**, which offers the two main aspects of **temporal sequence (the When)** and **actor-centric analysis (the Who)** (Sahide et al., 2020a,b). SPA advances beyond static power analysis by dividing the inquiry into three

separate yet connected chronological phases. This approach is particularly useful for examining dynamic, intervention-driven processes, such as those facilitated by YKAN, where power relations are continuously evolving.

The first phase, **Power Background**, sets out the historical and structural context that came before the intervention. It looks at the deep-rooted inequalities, institutional setups, and hidden conflicts that support current power dynamics. In the case of the Punan Batu, this includes mapping the historical patron-client ties with the Bulungan Sultanate and the legacy of state policies that have systematically marginalised nomadic ontologies.

The second phase, **Power Delivery**, forms the heart of agential action. This phase specifically examines the strategies, tactics, and negotiations used by particular actors as they actively navigate existing power structures. The focus here is on **who** is involved. It directs analytical attention toward actors, especially YKAN as a key intervener, but also includes community leaders, government officials, and corporate representatives. This phase scrutinises their capacity for action, strategic decisions, and how they utilise resources to alter, uphold, or challenge the status quo. It is during this phase that the framework most clearly highlights human agency within structural limitations.

Finally, **Power Adjustment** examines the outcomes and reconfigurations that follow intervention. This phase reveals how the actions taken during Power Delivery ripple through the social fabric, changing power relations in often unexpected ways. It tracks how different actors adapt, resist, or strengthen their power and identifies the emergence of new social structures, alliances, and conflicts. This phase answers not only how but when the consequences appear, as well as for whom they differ, exposing the winners and losers in the recalibrated political landscape. By focusing on both time and agency, SPA provides a strong narrative framework that captures the unfolding drama of power, setting the stage for other theoretical lenses to deepen the analysis.

2.2. The What: Power Cube as Typological Lens

While SPA provides the chronological framework, Gaventa’s (2006) Power Cube offers a comprehensive, multidimensional typology for analysing power relations. This framework looks at power across three interconnected dimensions. First, the **Faces of Power** distinguish its forms: **Visible Power** (observable in formal decisions, laws, and regulations), **Hidden Power** (controlling the political agenda and excluding issues from discussion), and **Invisible Power** (shape beliefs, perceptions, and preferences through ideologies and discourses). Second, the **Spaces of Power** identify where power is exercised: in **Closed Spaces**, where elites make decisions behind closed doors; **Invited Spaces**, where authorities invite certain actors to participate; and **Claimed or Created Spaces,** where marginalised groups assert their own autonomous arenas for action. Third, the **Levels of Power** span from the **Local** to the **National** and the **Global**, highlighting how power dynamics operate across different geographical and institutional scales.

Applying this integrated typology to each SPA stage allows us to systematically analyse not only how power functions through open coercion, hidden agendas, or ideological influence, but also where power is exercised and across which scales these processes occur during the recognition process. This multidimensional approach provides a more detailed understanding of the power relations that enable or restrict the recognition of the Punan Batu's customary forest rights.

2.3. The How & Why: Governmentality as the Underlying Logic

The third layer, Governmentality (Foucault, 1991; Li, 2007), enables the analysis of the underlying mechanism and rationality that explain how and why power relations function as they do. This perspective shows that the state's agrocentric bias is not just a policy preference but a form of rationality; a dominant way of thinking that favours sedentary, agrarian lifestyles and systematically problematises nomadic ways of life (Li, 2020). Additionally, it demonstrates that tools like participatory mapping and the formalisation of customary institutions are technologies of government. These are not merely neutral techniques but mechanisms that discipline fluid spatial practices and egalitarian social structures into forms legible to the state, thereby actively shaping the very "customary law community" they claim to recognise (Agrawal, 2005). In this way, Governmentality explains how both visible and hidden power techniques ultimately generate the effects of invisible power.

2.4. Analytical Integration: The SPA Cube Framework in Practice

This integration addresses specific analytical gaps in studying the Punan Batu case. Sequential Power Analysis alone captures temporal dynamics but misses power typologies. The Power Cube provides typology but lacks temporal sequencing. Governmentality reveals rationalities but obscures actor agency. The SPA-Cube thus enables tracing how power transforms across time, space, and consciousness simultaneously in this case study of studying the unique dynamics unfolding in the recognition of a nomadic community.

Together, they generate a comprehensive analytical approach, as outlined in Table 1. In this synthesis, the Power Cube serves as the analytical lens, classifying forms of power along the chronological sequence mapped by SPA, while Governmentality provides the deep-seated logic that drives the entire system. This integrated SPA Cube Framework is uniquely suited to examine the paradoxical outcomes of recognition processes, enabling us to trace how strategies of empowerment in the Power Delivery phase can, through governmental technologies, unintentionally discipline communities and reinforce the very logics of exclusion they aim to challenge.

Table 1. Analytical Framework for Power Dynamics: The SPA Cube Framework

**Analytical Dimension**	**Guiding Questions (Integrated Framework)**	**Power Cube Dimensions**	**Governmentality Concepts**	**Empirical Focus (Punan Batu Case)**
**POWER BACK-GROUND** *Historical & Structural Foundations*	How are historical and structural inequalities formed? What state rationality positions nomadic life as problematic?	**Faces:** Visible (concession permits), Hidden (historical patronage), Invisible (egocentric discourse) **Spaces:** Closed (colonial administration), Invited (resettlement programs) **Levels:** Local (sultanate), National (state policies)	Agrocentric rationality, Problematization of nomadism, Historical technologies of settlement	Patron-client relations with Bulungan Sultanate State-led "isolated community" discourse Timber and palm oil concessions

**POWER DELIVERY** *Agency & Strategies*	What strategies do actors use to change power dynamics? How do governmental technologies reshape subjectivities?	**Faces:** Visible (mapping), Hidden (informal networks), Invisible (identity formation) **Spaces:** Invited (participatory mapping), Claimed (Punan autonomous spaces) **Levels:** Local (district govt), National (ministry), Global (TNC/YKAN)	Technologies of legibility, Subject formation, Governmentality of recognition	Participatory mapping as territorialization Use of genetic research as epistemic capital Formalization of customary institutions Navigating bureaucracy through informal networks
**POWER ADJUST-MENT** *Outcomes & Responses*	How do power relations reconfigure post-intervention? What forms of resistance and adaptation emerge? Who benefits and who loses?	**Faces:** Hidden (corporate resistance), Invisible (internalized conflicts) **Spaces:** Closed (corporate decisions), Claimed (counter-conduct) **Levels:** Local (village conflicts), National (ministry verification), Global (conservation agendas)	Counter-conduct, Unintended effects, Subjectivity crises	Rejection from timber companies (2 of 3) Reproduction of patronage in new forms Internal conflicts within Punan Batu Emergence of counter-conduct strategies

[A detailed methodological protocol of this integrated SPA-Cube Framework, including its step-by-step analytical procedure, operational guidelines, and comprehensive analytical table, has been developed as a standalone methods article for wider application. This companion piece is being co-submitted to *MethodsX* (Elsevier) concurrently with this empirical analysis]

3. Methodology and Positionality

This study applies ethnographic methods to generate grounded data for the analysis of the power structures influencing the recognition process for the Punan Batu. This method is especially suitable because it goes beyond simple description to critically investigate how power relations - embedded in state policies, market forces, and historical patronage - systematically produce and sustain marginality (Li, 2007). Our approach aims to reveal not only the visible manifestations of power but also its hidden and invisible dimensions, along with the governmental rationalities that enable such power to operate.

3.1. Ethnographic Approach and Data Collection

Data collection was conducted over 36 months (2021-2025), combining participant observation, in-depth and walking interviews, and critical document analysis. This multi-method approach allowed for triangulation and a deep understanding of the power dynamics at play.

- **Participant Observation:** The first author lived alongside the Punan Batu community in multiple extended field visits, observing daily routines, community meetings, and interactions with external actors like YKAN staff, company representatives, and government officials. This immersion was crucial for grasping the subtle, often invisible, ways in which power is negotiated and internalised.
- **Walking Interviews:** Conducted while moving with Punan Batu members through their *adap* (living space), this method provided rich, spatially-grounded data on territorial practices, ecological knowledge, and the tangible impacts of the territorial squeeze.
- **In-depth Interviews:** We conducted 25 formal interviews with key informants, including Punan Batu elders and family heads (10 persons), YKAN field staff and managers (4 persons), district government officials from the Village Community Empowerment Agency (1 person), and heirs of the Bulungan Sultanate (1 person). Interviews focused on historical relations, decision-making processes, and perceptions of the recognition initiative.
- **Qualitative analysis of the media:** We analyzed 115 minutes 42 seconds of publicly available video documentation from diverse sources, including YKAN documentary (23:28), KOMPAS TV (24:13 + 3:49), NeT TV (18:50), CNN Indonesia (28:03), and BBC News Indonesia (17:19). These materials were examined as public performances of recognition politics, revealing how different actors frame and represent the struggle for different audiences.
- **Critical document analysis**: We conducted a systematic analysis of multiple document types to trace the evolution of power relations and state rationalities:
    - Historical Colonial & New Order Archives: Reports on the “Punan isolated tribal development project” (1964-65) and other resettlement programs, documenting the early problematization of nomadism.
    - Sultanate Documents: Handwritten letters and land claims from the heirs of the Bulungan Sultanate, used to assert territorial control.
    - Contemporary Policy Documents: The Bulungan Regency Regional Regulation on MHA Recognition, Minister of Home Affairs Regulation No. 52 of 2014, and related legal frameworks that form the agrocentric regulatory architecture.
    - Forest Governance Instruments: Maps and permits for the three Forest Utilisation Business Permits (PBPH) - PT ITCI Kayan Hutani, PT Inhutani, and PT Rizki Kacida Reana - and the oil palm plantation permit for PT Dharma Inti Sawit Lestari.
    - YKAN & NGO Internal Documents: Project proposals, meeting minutes, community agreements, participatory mapping outputs, and the final MHA verification report for the Punan Batu Benau Sajau Indigenous Community.
    - Media & Public Reports: Clippings from national media (e.g., CNN Indonesia, Kompas TV, BBC News, SEA Today News) covering the MHA recognition ceremony and the 2024 Kalpataru award, which were analysed as public performances of state recognition.
    - Scientific Publications: Genetic studies (Kusuma et al., 2023; Lansing et al., 2022) and anthropological reports used by YKAN as epistemic capital to legitimise the Punan Batu's uniqueness.

3.2. Operationalising the Analysis of Power and Temporal Dimensions

Guided by our SPA-Cube Framework, data were analysed to answer the *what, how,* and *why* of power across the three temporal sequences of the recognition process.

- **Temporal Dimension and Empirical Focus:** The analysis was structured into three phases of SPA, each with a distinct empirical focus. The **Power Background** phase (pre-2021) examined historical state formation, the legacy of the "isolated communities" program, and the

consolidation of patron-client networks. The **Power Delivery** phase (2021-2023) focused on the contemporary intervention, tracking YKAN's strategies, the participatory mapping process, and the formal verification for MHA recognition. The **Power Adjustment** phase (2023-present) analysed the aftermath of recognition, including emerging conflicts with companies and neighbouring villages, as well as the reconfiguration of patron-client relations.

- **Identifying Power (The What):** Within each temporal phase, data were coded according to Gaventa's (2006) typology. **Visible power** was determined in formal laws, concession permits, and direct conflicts. **Hidden power** was revealed in agenda setting, such as whose knowledge was prioritised in mapping or who was excluded from negotiations. **Invisible power** was analysed through discourses and internalised beliefs, such as the acceptance of agrocentric norms.
- **Unveiling Governmentality (The How and Why):** We examined how **technologies of government** (e.g., participatory mapping, MHA verification) functioned in each phase to make the Punan Batu legible to the state. We also analysed the **rationalities** behind these technologies, questioning why certain solutions (e.g., formalisation, settlement) were presented as common sense across different time periods.

3.3. Researcher Positionality and Triangulation

We explicitly recognise that the research process is fundamentally shaped by the researchers' positions (Maryudi & Fisher, 2020). This study leverages a unique triangulation of researcher positions to enhance the validity and depth of its critical analysis.

The first author was a staff member of YKAN during the research period, directly involved in facilitating the MHA recognition process. This insider status provided unparalleled access to meetings, communities, and internal documents, enabling a deep, emic perspective on the strategies and micro politics of intervention. However, it also necessitated continuous critical reflexivity to mitigate potential biases in interpreting data, particularly regarding YKAN's strategies and impacts.

The second and third authors, based outside Borneo, have researched Indonesian forest governance for over two decades. Their external, longitudinal perspective provided a crucial etic counterbalance, helping to situate the specific struggles of the Punan Batu within broader historical and structural patterns of power, conservation, and indigenous rights in Indonesia.

This strategic combination, which we term deep immersion paired with detached analysis, allowed for a robust triangulation of perspectives. Dialogues between the critical insider and reflective outsiders throughout the analysis helped to challenge assumptions, cross-checking interpretations, and ensuring that the findings were grounded in empirical reality while remaining critically sharp. This methodological rigour strengthens the credibility of our expose on the paradoxical nature of recognition politics.

4. Findings: The Three Sequences of Power in Punan Batu Recognition

4.1. Power Background: Historical Foundations of Marginalisation

Territorial Squeeze: The Multi dimensional Compression of Nomadic Space

The analysis of the power background shows that the marginalisation of the Punan Batu is a result of long standing structural processes rather than isolated contemporary phenomena. This has been built up through state power enforcing agrocentric logic, sultanate power sustaining and expanding patron client networks, and market power operating through commercial concessions and migrants, collectively producing what community members describe as "*being squeezed from*

all directions" (Akim Asdar, elder, field interview 2023). He explained, *"We love the forest, we love the food inside the forest. With this forest our lives depend; when there is no forest, we cannot live."*

4.1.1. State Rationality and the Problematization of Nomadism

State rationality systematically portrayed nomadism in what Foucault would describe as an *"abnormality"* requiring correction, constructing what Tania Li (2007) would call a *"problem space"* requiring state intervention. This agrocentric governmental logic is the invisible power shaping the entire system. The 1964-65 government survey for the 'Punan isolated tribal development project' aimed to develop "an orderly and systematic plan for the implementation of settlement programs" (Ring et al., 1964). This institutionalised approach regarded nomadism as a problem preventing integration into national development. A contemporary government official revealed the persistence of this agrocentric logic in 2023: *"We need to deliver the Christian religion, fixed addresses to provide healthcare, education, and development programs, such as tourism"* (Local government official, confidential, field notes, discussion 2023). This statement exposes how state bureaucratic requirements become invisible power, shaping what counts as "proper" citizenship. A contemporary development regulation illustrates the persistence of this rationality: *"The regional government will protect and empower the Masyarakat Hukum Adat (MHA) or Customary Law Community of Punan Batu MHA via government programs (as outlined in the acknowledgement decree of Punan Batu MHA)."* This rationality also aligns with YKAN's stated interventions following the decree: *"Firstly, the government and stakeholders will develop a protection action plan concerning their living space; secondly, livelihoods; and thirdly, basic services"* (YKAN Community Manager, 2023).

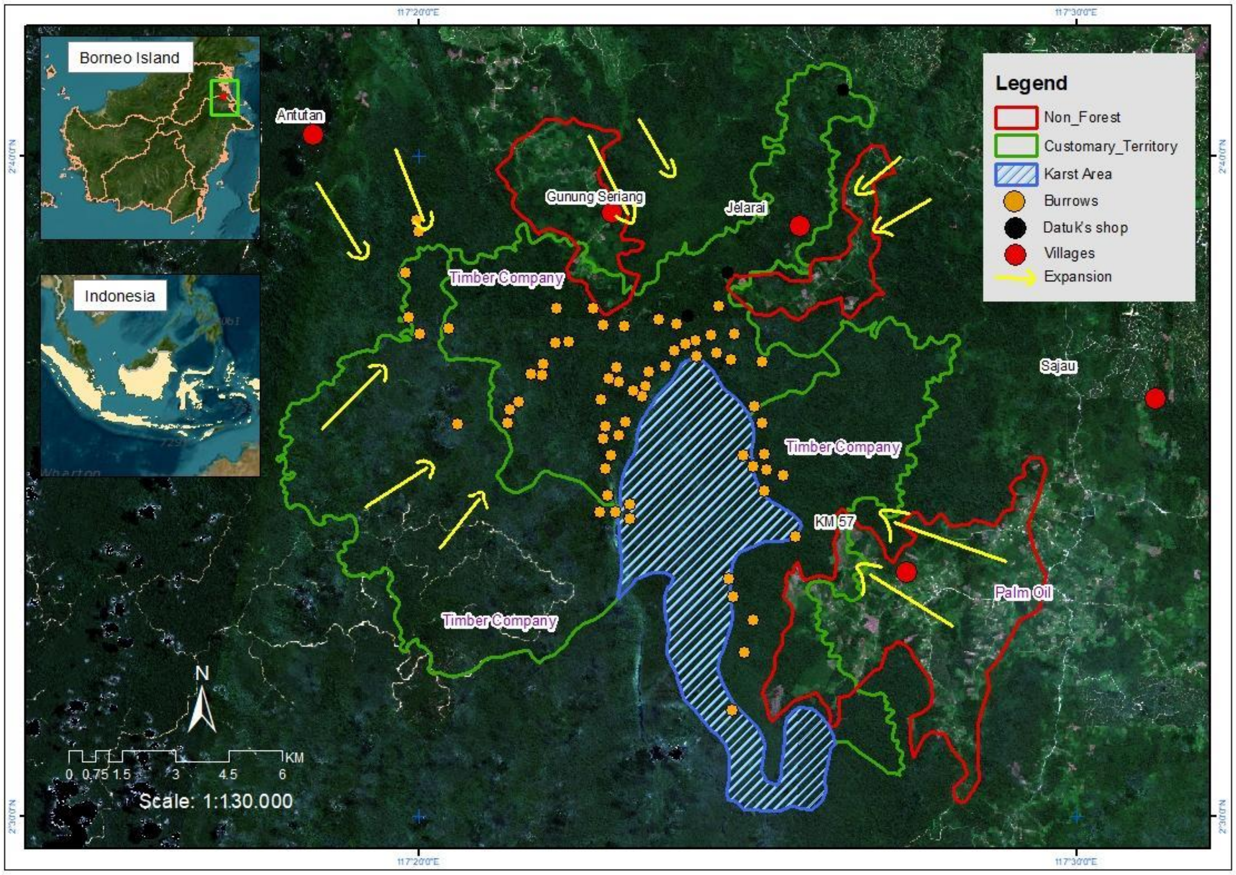

Figure 2. Multi-layered Territorial Compression of Punan Batu Customary Territory[1]

These government processes actively produced what Althusser might call "interpellated subjects". The Punan Batu were defined as "isolated communities," thereby compelling processes of transformation. A younger Punan Batu member described this subject formation process: *"Growing up, we internalised that we were backward, and isolated. It took genetic research and YKAN's work to help us see that we're not problems but unique"* (Rudi, Punan Batu youth, interview 2024). This identity was starkly reinforced by the state. The Head of the Social Services Office of North Kalimantan Province, during his visit to Punan Batu and while providing logistical support, declared, *"According to research, the Punan Benau community is the only group considered 'very backward' compared to others. Their lifestyle remains primitive; they rely on nature, hunting, and gathering in the forest. To the Secretary General and the Minister of Social Affairs: this is our community that needs the Ministry's attention so that social assistance can be better tailored to their needs."*

4.1.2. Multi layered Territorial Squeeze: Concessions, Patronage, and Settlers

The problematizing rationality materialised through visible and hidden power, enacting a physical and economic "squeeze" on Punan territory as depicted in Figure 2. State visible power manifested through systematic resettlement policies. The persistence of this power became starkly evident when the government built wooden housing units for the Punan Batu in the 1990s. Community members consistently refused to occupy them, with elder Akim Kamal explaining, *"They built these wooden boxes for us downstream, but how can we live separated from the forest that gives us life?"* (Akim Kamal, field interview 2023). Another community member articulated their ontological resistance more poetically: *"We are happy to live in burrows and forest huts because every flying animal like kukupu (butterfly), majani, birds, watch the falling leaves. We can see wide open spaces, we feel cozy, we can take a breath with pleasure. If we live in the government house, we are like a person tied with an iron rope."* (Akim Asdar, YKAN's documentation). Market power operated concurrently through commercial timber and palm oil concessions that fragmented their living space.

Simultaneously, the hidden structure of power operated through intergenerational patron client relationships that created what one observer called "invisible chains of dependency." Historical accounts confirm the origin story in which the Punan Batu became "Sikil Maulana" after transferring ownership of swiftlet nest caves. Contemporary economic dependency continues this pattern with intensified exploitation. A Punan Batu hunter described these brutal economics: *"I worked for three days cutting one cubic meter of meranti wood. When I bring it to Datuk Rahim, he gives me only 20 kilograms of rice, some sugar, cigarettes, and fuel. In the market, that wood sells for one million rupiah."* (Malik, interview 2023). Datuk Rahim describes his role differently,

[1] The map illustrates the multi-layered territorial compression confronting the Punan Batu community. Their customary territory (*adap*) appears as an archipelago of mobility patterns rather than a contiguous bounded area, reflecting nomadic spatial practices. Three timber concessions (PBPH) totaling approximately 379,545 hectares form a perimeter around their territory, while the oil palm plantation (3,366 hectares) blocks eastern movement routes. Migration corridors of agrarian communities from Sulawesi encroach from the south, and the historical influence zone of the Bulungan Sultanate heirs extends from the north. The Punan Batu's living space demonstrates high mobility patterns with multiple liang (caves) and lepo' (temporary shelters) scattered throughout the Gunung Benau area, representing adaptive responses to increasing territorial constraints. Critical resources like fruiting trees, tuber grounds, hunting areas, and honey sites show clustering in the increasingly fragmented forest patches between concession areas.*

as benevolent paternalism: *"Without my kiosk, they would starve during lean seasons. I provide everything on credit: rice, medicine, fuel. When the harvest is poor, I forgive their debts. The government never does this for them"* (Datuk Rahim, sultanate heir, interview 2025).

This relationship exemplifies *hidden power*: it operates not through law but through informal, socio economic dependency, controlling access to vital resources and markets. The sultanate's patronage did not just exploit; it actively disciplined Punan Batu mobility and economic autonomy, funnelling their labour and products into a captive, inequitable system. This created a vertical squeeze, compounding the horizontal squeeze from state concessions and migrant settlers, locking the community into a state of perpetual dependency that made them legible and manageable not only to the state but also to traditional elites.

The collision between national agrocentric policies and local nomadic realities created what James Scott (1998) might call "illegible spaces." A Ministry of Forestry official acknowledged this fundamental mismatch: *"Legal aspects such as recognition of Customary Law Communities from the Regency have been fulfilled, as well as Kalpataru award for Punan Batu. Other requirements, alongside customary forest management plans, must also be considered. Spatial understanding varies between nomads and other Indigenous communities. Their lifestyles cannot be equated with those of other farming groups. Nomads base their movements on resources, while States view land ownership differently. As a result, opinions on access differ."* (Integrated Team, Handling Tenurial Conflicts and Customary Forests, Ministry of Forestry, minutes of meeting, 2025).

4.1.3. Claimed Spaces and Everyday Resistance in the Forest

In response to exclusion from formal power spaces and the territorial squeeze, the Punan Batu maintained autonomous claimed spaces within the forest. Their sophisticated knowledge systems constituted what Michel de Certeau (1984) would call "tactics of the weak" everyday forms of resistance through spatial practices. Power operated through exclusionary closed spaces in which critical decisions were made without Punan Batu participation. The sultanate's control over swiftlet nest caves created what community members called "forbidden territories." As elder Akim Arif explained, *"We may have lived in these forests for generations, but certain caves became 'sultan's property' where we need permission to enter and harvest"* (Akim Arif, elder, interview 2023).

Within the forest, they exercised autonomy. The symbolic language (Belahiq) served as what community members called 'our forest signal.' As Rina explained, *"When we place certain leaf arrangements along trails, our family knows if there's danger ahead, if someone is sick upstream, or where we've found good hunting. It's a messaging system that outsiders cannot read"* (Rina, Punan Batu woman, walking interview 2023). The Menira praying song, in the Latala language, performed in huts, burrows, and caves in the forest, established sacred claimed spaces. Elder Akim Asdar described its importance: *"When we sing Latala in the forest, we are speaking with the forest spirits and reminding them that this is still Punan forest territory, and we ask why our forest is decreasing."* (Akim Asdar, elder, interview 2024). Their refusal to occupy state houses and preference for forest dwellings were thus profound acts of ontological resistance against the state's "sedentarisation imperative" and "technologies of power" like the Respen program documented by Sellato (2001).

This section has outlined the historical and structural roots of Punan Batu's marginalisation across multiple power dimensions. The analysis now moves to Section 4.2, which examines how YKAN navigated this complex power landscape through strategic interventions that both challenged and unintentionally reinforced these governmental logics.

4.2. Power Delivery: YKAN's Navigation Strategies

4.2.1. Agency and Visible Power Maneuvers

YKAN's Strategic Use of Scientific Research as Epistemic Capital

YKAN systematically transformed genetic research into powerful political leverage. Dr Pradipta Kusuma from the Eijkman Institute, whose team conducted genetic studies, explained their unexpected political usefulness: "*Our research aimed to understand human migration patterns in Borneo. We do not have the capacity to help protect their forest when Punan Batu ask for help. So, we navigate our research to identify what can be employed for conservation. We found their distinct genetic markers separating them from agricultural Dayak communities, which became scientific evidence of their unique status as Borneo's last active hunter-gatherer… We also studied the frequency of movement as nomadic activity using GPS and identified their home range territory*" (Dr Kusuma, 2023).[2]

A YKAN manager explained how they used this science: "*The Punan Batu area is important because it is inhabited by a community that, according to research, has original DNA and is considered the last active hunter-gatherer group, consisting of about 35 families that live in harmony with the forest. They also align with conservation principles, aiming to show the world that this way of life is what future human civilisation needs to thrive.*"[3] *The next step is protection. We hope that, by the end of this journey, the community will have sovereignty over their lands so they can continue to live their unique way of life, which relies heavily on the forest. They are the true guardians of the forest."*[4]

One Regent remarked, *"Of course, this has potential for Bulungan Regency if it can succeed, with the emphasis that the originality and local wisdom present in this area, especially Punan Batu, continue to be preserved, including two aspects related to territory that are maintained and protected.* (Regent of Bulungan, 2023)[5]. The studies by Kusuma et al. (2023) showing the Punan Batu's ancestral origins from mainland Asia became what staff called "an academic trump card" in policy discussions.

Formal Negotiation Tactics with District Government

YKAN's bureaucratic entrepreneurship was demonstrated through careful preparation of what officials called "turnkey policy packages." A DPMD official explained the process: *"YKAN didn't just bring problems; they brought complete solutions - draft regulations, mapped territories, scientific justification, and even budgetary calculations. They supported the Punan Batu for recognition."*

YKAN used persuasive power that was often more effective. YKAN's negotiation strategy included systematic follow-up mechanisms, with one staff member noting, *"We tracked every document through the bureaucracy, gently reminding officials at each stage, informally discussing*

---

[2] https://www.youtube.com/watch?v=wbIEkkLRfRY&t=1115s Kompas.com Youtube. "Kisah Ilmuwan Meneliti Punan Batu, Pemburu dan Peramu Terakhir di Kalimantan" , accessed on 12 October 2025

[3] https://www.youtube.com/watch?v=vMcuyc3Xrfg&rco=1 Net Newsroom Youtube [KISAH NYATA] KONDISI WARGA PUNAN BATU, SUKU DAYAK ASLI PEDALAMAN BORNEO... , accessed on 12 October 2025

[4] https://www.youtube.com/watch?v=8IwqWNlcltQ&t=1476s CNN Indonesia Youtube. "Hikayat Suku Punan Batu Benau Sajau" , accessed on 12 October 2025

[5] https://www.youtube.com/watch?v=vMcuyc3Xrfg&rco=1 Net Newsroom Youtube [KISAH NYATA] KONDISI WARGA PUNAN BATU, SUKU DAYAK ASLI PEDALAMAN BORNEO... , accessed on 12 October 2025

with government officials over coffee or dinner, and also with the Secretary of Regent who can give directions to government officials. We trained the Customary Committee to do verification, and facilitated visits to the Punan area. It became impossible for them to lose or ignore the case" (YKAN staff, discussion 2025).

Participatory Mapping as Both Empowerment and Territorialisation

The mapping process revealed fundamental ontological conflicts between state and nomadic spatial conceptions. A YKAN staff member explained the difficulty: "*The Punan Batu understands territory as a network of relationships - hunting trails, fruiting seasons, forest tubes for food, and cave networks. We had to translate this living geography into the static polygons recognised by forestry regulations.*" (YKAN staff interview 2025).

YKAN staff recognised this territorialisation as a necessary compromise: "*We knew we were freezing their fluid reality into cartographic snapshots, but without these state-legible boundaries, their territory would stay unseen by the law*. (YKAN staff, interview 2025).

4.2.2. Navigating Hidden Power Networks

Informal Alliances with Sultanate Heirs as Cultural Brokers

YKAN's pragmatic engagement with existing power structures involved strategic co-optation of traditional authority. Datuk Rahim, the primary sultanate heir, articulated his indispensable role: "*All parties (government officials, media, and researchers), including YKAN, always contact me to help them connect with the Punan Batu community. I arranged their transport and met them at Punan Batu. I am also an interpreter between them. YKAN always invites me to speak about the Punan Batu at meetings with the government."* (Datuk Rahim, interview 2025)

YKAN's staff explained the calculated decision: "*We conducted a power mapping that showed that Datuk Rahim controlled access to the Punan Batu. Instead, we made him our ambassador, providing incentives that aligned his interests with ours."* (YKAN staff, confidential discussion 2025). These incentives included infrastructure development at his base camp and maintaining his role as the primary liaison.

Behind-the-scenes Lobbying of District Officials

YKAN used what local officials called 'coffee shop diplomacy' to bypass bureaucratic delays. A district government officer said, *"Formal meetings will be the official space to complete strategic and documented matters. But frankly, informal meetings like coffee help us in the recognition process, allow us to explore issues and context more freely when understood technically, and help us understand perceptions more comfortably because there is no pressure. Many complex things are easier to understand during informal meetings."* (Officer from Community and Village Empowerment Agency, interview 2025).

One YKAN staff member described their approach to building coalitions: *"We identified the real decision-makers in each agency - often not the directors but the section heads who actually draft policies. We built personal relationships with them, understanding their career and showing how supporting the Punan Batu could help achieve them"* (YKAN staff, discussion 2025).

Strategic Exclusion of Certain Actors from Negotiations

YKAN deliberately curated participation to maintain control over the process. A staff member admitted: "*We work with NGOs in other areas of Bulungan that are experienced in facilitating the recognition of customary communities (masyarakat adat). For Punan Batu, we directly facilitated the process at the site and regency government levels. We use a science-based approach and*

persuasive power to navigate and influence government policy. This approach differs from that of advocating NGOs or idealistic academic researchers who use confrontation." (YKAN Officer, confidential interview 2025).

This selective inclusion also applied to community representation. Another staff member remarked: *"We worked primarily with Punan Batu elders who understood the need for compromise. We are not yet working with women's groups and youth members for meetings with the government, who might have taken tougher positions against the sultanate or companies"* (YKAN staff, interview 2025).

4.2.3. Reshaping Invisible Power through Discourse

Reframing Punan Batu from "backward" to "last nomads"

YKAN orchestrated a broad discursive shift that changed the community's political identity. A YKAN officer explained their approach: *"We systematically replaced the term 'masyarakat terasing' [isolated community] with 'komunitas nomaden terakhir' [last nomadic community] in all documents, media and discussions.* The impacts of this reframing became evident in official statements. The Bulungan Regent stated during the MHA recognition ceremony: "*The Punan Batu people have not been touched by government, are 'relatively backward', and today they are people of Bulungan who must receive support, especially '*masyarakat adat*' of Bulungan Regency, with the notion of originality, local wisdom, especially the importance of protecting Punan Batu to continue to exist*." (Regent's Speech, April 2023).[6]

Using Genetic Research to Establish Political Legitimacy

The scientific research became what one policy analyst called 'biological citizenship' evidence. A government official noted how this changed bureaucratic perceptions: *"The DNA evidence transformed the discussion from whether they deserved recognition to how quickly we could recognise them. It provided the objective proof that bureaucrats need to justify unconventional decisions"* (Regency government official, 2023).

YKAN framed the anthropological research by Lansing and Kusuma (2023) on Punan Batu mobility patterns using GPS trackers as evidence of their unique nomadic system, as living space, and cultural heritage worth protecting.

Internal Conflicts within Punan Batu over New Identities

The process of identity reconstruction created considerable internal tension. Younger Punan Batu member Rudi shared his ambivalence: *"Now we have been recognised as '*masyarakat hukum adat*' by every visiting official or journalist. They expect to see blowpipes and bark clothing. But we also want smartphones and education. Are we allowed to be both traditional and modern?"* (Rudi, Punan Batu youth, interview 2023).

Another Punan member acknowledged, *"We, Punan cannot live out of the forest, but need special attention from government. But if the service is provided, Punan can accept both; protect the forest and follow modernisation."* (Safar, interview 2022).[7]

---

[6] https://www.youtube.com/watch?v=vMcuyc3Xrfg&rco=1 Net Newsroom Youtube [KISAH NYATA] KONDISI WARGA PUNAN BATU, SUKU DAYAK ASLI PEDALAMAN BORNEO... , accessed on 12 October 2025

[7] https://www.youtube.com/watch?v=GbHhon0sKvQ BBC News Indonesia, Youtube. "Suku pemburu-peramu Punan Batu: Bertahan di hutan atau bertarung melawan zaman - BBC News Indonesia", Accesed on 12 October 2025

4.2.4. Multi-level Power Navigation and Organizational Positioning

YKAN's multi-level navigation reflects complex organizational interests that extend beyond conservation targets to include funding sustainability and institutional reputation. The organization's dependence on international philanthropic funding creates pressure to demonstrate measurable success, incentivizing technical solutions that produce tangible outputs for donor reporting while building organizational credibility.

Local Navigation: Building District Government Support

YKAN's local strategy created what staff termed 'ownership through benefit-sharing.' A field manager explained: *"We designed the recognition process to deliver clear political wins for local officials - media coverage, awards, and national attention. We made supporting the Punan Batu synonymous with progressive leadership"* (YKAN Manager, fieldnoted 2024). This approach produced tangible results with the Bulungan Tourism Agency noting: *"YKAN showed us how the Punan Batu could become our tourist attraction with Geo Park rather than our development problem."* (Head of Bulungan Tourism Agency, field notes and discussion, 2023). These strategies served a dual purpose: advancing community rights while securing YKAN's institutional positioning.

National: Engaging Forestry Ministry Protocols

At the national level, YKAN positioned the case as an opportunity for policy innovation. YKAN's national policy coordinator explained their strategy: *"We framed this as a test case for implementing the Constitutional Court's ruling on indigenous rights. We provided the ministry's verification team with information on the uniqueness of Punan Batu as nomadic communities"* (YKAN Policy Coordinator, discussion, 2025).

A Head of Integrated Team for Customary Forest Verification and an academic from Diponegoro University explained: "*Nomadic groups have a different understanding of spatial patterns than other indigenous communities. Recognising MHA and Customary Forest Rights is a step in the right direction. Their lifestyle cannot be compared with agrarian communities."* (minutes of meeting on the proposal for the Punan Batu Customary Forest, 2025).

A senior official from the Ministry of Forestry described, after visiting Punan Batu: "*Indeed, our team wants to help Punan Batu because today we verified or will protect the customary forest or place vital for the lives of Punan Batu, such as animals, tubers, and other sources of food. What is needed is in the Menira' (pray song). We will continue this need (that cites in Menira') to Jakarta, to the leader and also the whole office that will give protection to the forest."* (field visit, technical verification, 2025).

Global: Leveraging TNC Networks and International Funding

YKAN strategically linked local struggles to global conservation agendas. *Punan Batu is seen as representing both cultural preservation and forest conservation—precisely what international donors seek to support. They adapted their nomadic practices into Indigenous Forests* (Hutan Adat) *as part of YKAN's social forestry goals in Indonesia* (YKAN staff, 2025).

This global framing attracted significant resources. TNC networks provided YKAN with access to donors who recognised that supporting indigenous rights was the most cost-effective conservation strategy. YKAN secured funding that local NGOs could never access because YKAN is directly affiliated with TNC, an international conservation NGO and donor that supports YKAN's indigenous-led conservation. The multi-level navigation created what one observer called

‘a perfect policy storm’: local political interests aligned with national policy innovation and global conservation funding, temporarily overcoming the structural barriers that typically marginalise nomadic communities.

4.3. Power Adjustment: Paradoxical Outcomes and New Configurations

4.3.1. Visible Power Reconfigurations

Formal MHA Recognition Versus Ongoing Territorial Conflicts

The official recognition of Punan Batu as *Masyarakat Hukum Adat (MHA),* as set out in Bulungan Regent Decree No. 188.45/319 in April 2023, created a stark paradox. Although the ceremony included celebratory speeches and media coverage, the reality on the ground was still filled with conflict. Elder *Akim* Asdar highlighted this contradiction: *“They gave us this beautiful certificate in a big ceremony, but the next week, the company and settlers’ bulldozers were still clearing our forest. The paper recognises our existence, but it cannot stop the destruction. We want to give back this certificate decree”* (*Akim* Asdar, elder, interview 2025).

The territorial conflicts intensified precisely because of the recognition. A letter from PT ITCI Kayan Hutani to the Ministry of Forestry clearly stated: *“PT ITCI Kayan Hutani respect the government's recognition of Punan Batu, but our concession permits were issued first, remain valid and require us to implement our business work plan. We cannot relinquish productive forest areas based on* Hutan Adat.*”* (Document, 2025).[8] This confrontation between legal instruments revealed the limitations of MHA recognition without corresponding territorial protection.

Company Rejections of Customary Forest Proposals

The technical verification process for the Customary Forest proposal revealed organised corporate resistance. Two of the three concession holders formally rejected the proposal. A PT Rizki Kacida Reana representative stated: *“Our work area is within the Punan Batu customary law community area of 2,761 hectares, which is located in the 2nd period of our business work plan or Rencana Kerja Usaha (RKU), so if it is released, it will disrupt the company's work plan.”* (Company Representative, minutes of meeting 2025).

The North Kalimantan Forestry Agency official responsible for overseeing the process acknowledged the deadlock: *“We are caught between legal concession rights and constitutional recognition of indigenous rights. The companies have valid permits, while the Punan Batu have legitimate historical claims. There are no simple solutions”* (Forestry Agency Official, interview 2025).

Kalpataru Award as a New Form of State Recognition

The 2024 Kalpataru Award for environmental preservation presents yet another paradoxical form of recognition. While honouring the Punan Batu as ‘environmental guardians,’ it simultaneously reinforces state authority to define and reward ‘proper’ environmental subjectivity. As one Ministry of Environment and Forestry official noted during the award ceremony: “*Their tradition, which has been passed down for thousands of years and requires them to preserve the forest, was our primary consideration in awarding this Kalpataru. They are like hotspots that we need to continue to foster so they can ignite other communities and inspire them”*.

---

[8] Response letter regarding the determination of customary forests to the Director of Tenurial Conflict Management and Customary Forests, Ministry of Forestry

(MoF Official, award ceremony 2024).[9]

However, the Punan Batu elder, Asdar, expressed ambivalence: *“Now we are called ‘environmental heroes’ while our forest is being destroyed. The award feels like a consolation prize for our suffering”* (*Akim* Asdar, Punan Batu elder, interview 2025).

4.3.2. Hidden Power Adaptations

Patron Client Networks Reproducing in New Forms

The recognition process inadvertently strengthened Datuk Rahim's position as a necessary intermediary. He now serves as the official translator and liaison during government meetings, reinforcing his role as a gatekeeper. *“The officials and YKAN need me more than ever,”* Datuk Rahim noted with satisfaction. *“Without my translation and explanations, they cannot understand the Punan Batu ways”* (Datuk Rahim, interview 2025).

The economic dependence also took new forms. A Punan Batu hunter explained: *“Now, when Punan need to attend government meetings, Datuk Rahim contacts them and assists with transportation. YKAN covers all costs for Datuk Rahim, including transport and expenses during the two-day stay in the city. The new patron is added to the existing ones.”* (YKAN staff, interview 2025).

Emergence of Internal Punan Batu Elites

The formalisation process created new internal hierarchies within the traditionally egalitarian community. Safar, the appointed RT head who had begun farming, gained disproportionate influence as the ‘literate representative.’ Younger members noted this shift with concern. “*Safar now speaks for all of us in meetings, but he lives differently from us. He farms, sells land, and thinks like a settled person,”* observed one nomadic family head (Anonymous, Punan Batu member, interview 2024).

Continued Exclusion Through Bureaucratic Technicalities

The verification process for the Customary Forest proposal introduced new forms of bureaucratic exclusion. The requirement for "clear and uncontested boundaries" became an impossible standard for a nomadic community. A Staff member of the Forestry Department and verification team admitted: *“Their territory claims overlap with three active concessions and migrant settlements. The Forestry Department received a report on land sales and purchases that requires attention to prevent social conflict. Accelerating the designation of customary forests is urgent for the Punan Batu indigenous community. We need unambiguous boundaries for the Customary Forest decree, but their way of life resists such precision”* (Staff of North Kalimantan Forestry Agency, minutes of meeting 2025).

4.3.3. Invisible Power Transformations

Identity Crises Among Punan Batu Youth

The reconstruction of Punan Batu identity from ‘backward’ to ‘last nomads’ has caused generational tensions. Younger members like Safar expressed mixed feelings: *“Indeed, we Punan Batu cannot leave the forest, but I imagine there must be a special treatment from the government, how to teach and help Punan Batu to continue living in the forest, because Punan Batu do not*

[9] https://www.ykan.or.id/id/publikasi/artikel/siaran-pers/legalitas-hutan-adat-masyarakat-punan-batu/ accessed on November 19, 2025.

want to be removed from the forest, but we also want services in the area of Punan Batu. We will accept both: Punan still maintain their forest territory, but also participate in modern life." (Safar, Punan Batu youth, interview 2022).

The performance of 'authentic' nomadism for external audiences added extra psychological burdens. A young Punan Batu woman confessed: *"When people from the city (government and journalists visit), we must wear bark clothing and demonstrate 'traditional' activities. It feels like we are performing our own culture as a spectacle"* (Anonymous, Punan Batu woman, discussion 2024).

A Punan Batu youth spoke as a form of counter-conduct. *If there is a teacher, what would you learn? He answered, "laws and regulations," the reason being that we will keep growing. Currently, the Punan Batu are still being fooled and deceived by people; for instance, some parties offer to guard our forest, but it is sold to someone else. I have an ambition to protect the forest.* (Ali, 2023)[10]

Erosion of Traditional Knowledge Through Formalisation

The translation of oral traditions into written regulations began to alter the process of knowledge transmission. *We cannot read the map in the decree, we just know the places to find food, animals, honey and caves."* (*Akim* Asdar, 2024).

The formalisation of customary institutions also altered decision-making processes. *"Before, we discussed until everyone agreed. Now we have 'leaders' and intermediaries who make decisions based on what the NGO or government wants,"* noted a community member (Anonymous, Punan Batu member, interview 2024).

New Dependencies on NGO Intermediaries

YKAN's extensive involvement created what one staff member called 'the NGO trap.' As the YKAN field officer acknowledged, *"We wanted to build their capacity, but they've become dependent on our facilitation. They wait for us if they face a problem; they report to Datuk Rahim. So, YKAN and Datuk Rahim help them to prepare documents, and even transport them to events."* (YKAN Field Officer, interview 2025).

This dependency extended to knowledge production. A Punan Batu elder admitted: *"We cannot read the maps and documents about our own territory. We need Datuk Rahim and YKAN to explain what they have written about us"* (*Akim* Arif, elder, interview 2024).

4.3.4. Spatial and Level Reorganisations

Creation of New "Invited Spaces" in Verification Processes

The Customary Forest proposal process established formal 'invited spaces' where Punan Batu representatives could speak, but under very constrained conditions. A community member described the verification meeting: "*We sat at the big table with officials, but they used so many technical terms and legal phrases. We could only speak when they asked us questions, and even then, our answers had to be translated and simplified by Datuk Rahim*" (*Akim* Asdar, Punan Batu, discussion 2025). These spaces remained tightly controlled. As a government official noted: *"We need their participation to legitimize the process, but the discussions must follow bureaucratic protocols and legal frameworks, We need to discuss with timber companies, neighboring villages,*

---

[10] https://www.youtube.com/watch?v=vvlFzFQWY9Y Kompas Youtube, "Cerita Layis, Gen Z dari Punan Batu, Pemburu dan Peramu Terakhir di Indonesia", accessed on 12 October 2025

and Director General of Sustainable Forest Management, Ministry of Forestry." (Verification Committee Member, minutes of meeting, 2025).

Loss of Traditional "Claimed Spaces" Due to Mapping

The cartographic formalisation of territory began to diminish the community's autonomous claimed spaces. The caves, forest, food, and animals, as *adap* (living space) used for nomadic activities, once known only through oral tradition, now appear on official maps. Elder *Akim* Asdar expressed concern: *"Now that the government recognises these places, they are no longer just our* adap *(living spaces) sites. They will become a 'Geo Park' that others can study and visit, but the forest also continues to decrease over time."* (*Akim* Asdar, elder, interview 2024).

The mapping also fixed previously fluid access arrangements. *"Before, we moved according to the seasons and animal movements for hunting. Now we experience that 'our territory' is squeezed every year by companies and settlers, and the map and decree cannot prevent it* (Asdar, Punan Batu hunter, discussion 2025).

Cross-Scale Alliances Between Local Communities and Global Actors

The struggle drew unexpected alliances across different levels. International conservation groups started using the Punan Batu case in global forums. A YKAN representative said, *"The Punan Batu represents the perfect convergence of indigenous rights and forest conservation. Their case helps us demonstrate that protecting traditional lifestyles is an effective indigenous-led conservation"* (YKAN staff Rep, interview 2025).

Simultaneously, the Punan Batu case exemplifies a global pattern highlighted in international indigenous rights discourse. A 2024 report by the UN Special Rapporteur on the rights of indigenous peoples identifies mobile indigenous peoples, including hunter-gatherers like the Punan Batu, as facing particular challenges because state legal systems are predominantly designed for sedentary and agricultural societies (Cali Tzay, 2024).

This multi-scalar attention created both opportunities and complications. As a YKAN staff member observed: *"The international spotlight gives us leverage with the national government, but it also raises expectations and simplifies complex local realities"* (YKAN staff, interview 2025).

The power adjustments documented in this section reveal the profound and often paradoxical consequences of recognition processes. While creating new political openings, they also generated new forms of dependency, internal stratification, and cultural transformation, demonstrating that empowerment and governmentality often go hand in hand.

5. Discussion: Interrogating the Paradoxes of Recognition

The integrated SPA-Cube Framework reveals that the recognition process for the Punan Batu constitutes a complex terrain where power operates dynamically across multiple dimensions, producing paradoxical outcomes that simultaneously empower and discipline the nomadic community. To systematically trace these power dynamics, Table 2 synthesizes how visible, hidden, and invisible power configurations shifted across the three sequential phases of recognition, while also illuminating the governmental effects that transformed empowerment strategies into technologies of subject formation

Table 2. Power Dynamics Across Recognition Sequences: Paradoxes and Transformations

SPA Sequence	Visible Power Dynamics	Hidden Power Adaptations	Invisible Power Transformations	Governmentality Effects
**POWER BACKGROUND** *Historical & Structural Foundations*	State resettlement policies; concession permits; territorial squeeze	Sultanate patron-client networks; debt bondage; economic dependency	Agrocentric discourse; problematization of nomadism; "backward community" narrative	Formation of "isolated community" subject; normalization of sedentary ideals
**POWER DELIVERY** *Agency & Strategies*	Participatory mapping; MHA recognition; formal ceremonies	Informal lobbying; strategic alliances with elites; exclusion of dissent	Reframing as "last nomads"; scientific legitimization; identity reconstruction	Discipline through legibility technologies; institutional formalization
**POWER ADJUSTMENT** *Outcomes & Responses*	Company rejections; Kalpataru award; ongoing conflicts	Patronage reproduction; internal elite emergence; bureaucratic barriers	Identity crises; new NGO dependencies; knowledge erosion	Counter-conduct; unintended subject formation; resistance adaptations

5.1. The Agrocentric Straight jacket: When State Legibility Erases Nomadic Ontology

The struggle for Punan Batu recognition exposes fundamental tensions between nomadic ontologies and state territorial logic. Our findings demonstrate that the state agrocentric framework, while ostensibly neutral, systematically invalidates non-sedentary ways of being. As articulated by Punan Batu elder *Akim* Asdar, *"The forest is not something we own. We are part of the forest, and it is part of us. We put up boundary signs to prevent outsiders from encroaching on the forest. Hopefully, our small forest, damaged by logging and a plantation company, will be protected for our small community. We would be grateful and also need the government's support."*[11] However, this condition is paradoxical; government regulations must also set boundaries as a condition for recognising customary areas. This ontological conflict resonates with James Scott's concept of state legibility, where complex social realities are simplified to make them administrable (Scott, 1998).

The participatory mapping process, intended as a tool for empowerment, functioned as a form of governmentality that disciplined fluid spatial practices into fixed cartographic representations. When Punan Batu hunters explained, "*Determination of customary areas through maps on a regent's decree does not help to protect the forest. Trees, hunting areas and gathering forest tubers are decreasing*," they expressed the violence of what anthropologist Tim Ingold calls the cartographic illusion, the reduction of lived space to bounded territory (Ingold, 2000).

The three-zone compromise negotiated by YKAN, while pragmatically necessary, exemplifies what Ferguson and Tania Li might describe as the anti-politics machine at work, transforming political claims about territory and rights into technical solutions such as zoning and mapping (Li, 2007; Ferguson, 1996). This process produces what we call the recognition paradox: communities must first deny their nomadic identity to become legible to the state, only to then reclaim that identity through state-sanctioned categories (Scott, 1998).

---

[11] https://www.youtube.com/watch?v=KI6q4wnVBl8 , YKAN youtube, "Punan Batu, Sang Penjaga Rimba", accessed on 12 October 2025

5.2. The NGO as Governmentality Broker: Empowerment as Discipline

YKAN navigation strategies illustrate the complex positioning of conservation NGOs as what we consider governmentality brokers - actors who both challenge and reproduce state rationalities. The organisation's strategic use of genetic research as epistemic capital shows how scientific knowledge can be weaponised in political struggles. As Dr Kusuma noted, "*Our research aimed to understand human migration patterns, and navigate them so that organisations can use them.*" YKAN recognised its power to legitimise the Punan Batu's unique status. This strategic essentialism, while effective, risks epistemic violence by simplifying complex cultural identities into biologically determined categories (Sylvain, R., 2014).

The formalisation of customary institutions exemplifies what Foucault would call subjectification, the process by which individuals are turned into subjects acknowledged by power structures (Foucault, 1991). When Punan Batu youth Rudi said, "*Now we have customary officials like the government wants, but our elders used to lead because of their wisdom, not because of titles,"* he pointed out how state recognition involves creating hierarchical structures that are foreign to traditionally egalitarian nomadic societies. YKAN's dual role as both challenger and reproducer of state logic illustrates what David Mosse identifies as the paradox of participation: how participatory approaches often end up reinforcing the very power structures they seek to transform (Mosse, 2004). The organisation's success in securing MHA recognition came at the cost of disciplining Punan Batu social organisation into state-compatible forms.

5.3. The Resilience of Patronage: Why Structural Power Persists

The persistence of patron-client ties despite formal recognition challenges the idea that legal rights automatically fix structural inequalities. As Datuk Rahim confidently stated, *"These NGO people understand that without me, and without our help and approval, they cannot reach the Punan Batu."* He demonstrated how traditional power structures adapt and absorb new governance arrangements.

The economic dependencies that persisted despite formal recognition, where Punan Batu hunters continued trading valuable timber for minimal rice rations, illustrate what Philip Abrams would describe as the distinction between the state idea and the state system (Abrams, 1988). While the state's notion of recognising indigenous rights promised change, the day-to-day operations of the state system replicated existing inequalities.

This resilience of patronage networks supports James Scott's theory of everyday forms of resistance and accommodation. Rather than dramatic confrontation, both patrons and clients engage in mutual adaptation that preserves the relationship's core inequalities while allowing for surface-level changes. As one Punan Batu hunter noted, *"We are grateful to accept the recognition decree and new structures because we need the forest to survive. But in our hearts, we remain people of the forest, a nomadic people in the forest."*

5.4. Multi-level Governance: The Scaling of Power Struggles

The Punan Batu case exemplifies what political geographers call the politics of scale, demonstrating how power struggles operate across different geographical levels. YKAN's strategic move from local to global arenas shows the boomerang pattern described by Keck and Sikkink, where local actors bypass obstructive national governments by appealing to international allies (Keck & Sikkink, 1998). The international attention generated by genetic research and conservation networks creates what we call scale leverage, using global recognition to influence local authorities. As a YKAN staff member observed, *"The international spotlight gives us*

leverage with the national government, but it also raises expectations and simplifies complex local realities." This scaling strategy, while effective, risks what Li calls rendering technical, reducing complex political struggles to manageable policy problems (Li, 2014)

The cross-scalar alliances also created new forms of dependency. As a Punan Batu elder admitted, *"We cannot read the maps and documents about our own territory. We need YKAN and Datuk to explain what they have written about us."* This illustrates what Mohan and Stokke identify as the localisation paradox, how global support for local empowerment can create new forms of external dependency.

5.5. Beyond Recognition: Toward Nomadic-Centric Governance

The Punan Batu experience highlights the need for what we call nomadic-centric governance, frameworks that start from and accommodate nomadic ontologies rather than suppress them. This involves moving beyond what Elizabeth Povinelli describes as the cunning of recognition, in which marginalised groups must perform authenticity according to dominant norms to access rights (Povinelli, 2002). The development of internal differences within Punan Batu, between nomadic and semi-settled members, exemplifies what Anna Tsing refers to as friction—the complex and unequal interactions that define globalisation. Instead of viewing this differentiation as a cultural loss, we can see it as a strategic adaptation within limited options.

Ultimately, the Punan Batu case calls for rethinking recognition beyond what Nancy Fraser describes as the redistribution recognition dilemma (Fraser, 2000). For nomadic communities, genuine recognition requires transforming the core categories through which states perceive and manage territory and mobility. As Punan Batu elder *Akim* Asdar reminded us, "*The papers are tools, not truths. Our real law remains in the forest."* This indicates that meaningful recognition must embrace legal pluralism and multiple territorialities instead of trying to fit nomadic worlds into sedentary frameworks (Riyanto, 2025).

The theoretical contribution of this analysis lies in showing how the SPA Cube Framework clarifies the dynamic interaction between different types of power over time. By exploring how visible, hidden, and invisible power operate through governmental technologies across various spaces and scales, we better understand why recognition can often lead to paradoxical results that both empower and restrain marginalized communities.

6. Conclusions

This study shows that the recognition process for Indonesia's last nomads presents a profound paradox. The mechanisms meant to empower the Punan Batu also regulate their nomadic way of life into forms that the state can understand. Our SPA Cube Framework reveals how power operates differently over time, with government tools turning empowerment efforts into means of shaping individuals.

Theoretically, this research advances environmental governance studies by highlighting the complex interplay among power, recognition, and resistance. We show how well-meaning conservation efforts can unintentionally strengthen the very agrocentric ideas they aim to oppose. The ongoing presence of patron-client networks, despite formal recognition, reveals the limits of rights-based strategies in changing deep-rooted inequalities.

Practically, our findings provide essential insights for policymakers and conservation practitioners. Firstly, current recognition paradigms need a fundamental rethink to include non-agrarian ways of being. Secondly, NGOs should adopt more reflexive approaches that recognise

their role as brokers of governmentality. Thirdly, effective intervention requires addressing existing power structures rather than bypassing them.

The Punan Batu case highlights a wider global issue: how to secure rights for mobile communities within mostly settled governance systems. Their struggle reminds us that real recognition requires not just legal inclusion but also ontological pluralism. As the Punan Batu elders assert, their genuine law remains in the forest, not in the documents that try to contain it. This shows that conservation and indigenous rights efforts must start from nomadic worldviews instead of forcing them into state-compatible frameworks.

Future research should examine comparative cases of non-agrarian community recognition across various political contexts. Long-term studies following the intergenerational effects of recognition would further clarify how governmental technologies influence community subjectivities over time. Ultimately, securing rights for nomadic peoples requires governance innovations that respect mobility as a valid way of life rather than a problem to be addressed.

Acknowledgements:
This paper completed with the assistance from various parties such as the critical feedback from colleagues in the Forest & Society Research Group (FSRG) UNHAS and the research funding support from the Hasanuddin University research grant.

Reference

Abdullah, A., Fisher, M. R., & Sahide, M. A. K. (2024). Environmental governance challenges of indigenous forest recognition: Climate solution ideal and its uneven outcomes in Indonesia. *Forest and Society*, 8(2), 402–421.

Abrams, P. (1988). Notes on the difficulty of studying the state. *Journal of Historical Sociology*, 1(1), 58-89.

Acciaioli, G. (2007). From customary law to indigenous sovereignty: Reconceptualizing masyarakat adat in contemporary Indonesia. In J. S. Davidson & D. Henley (Eds.), *The revival of tradition in Indonesian politics: The deployment of adat from colonialism to indigenism* (pp. 295-317). Routledge.

Agrawal, A. (2005). *Environmentality: Technologies of government and the making of subjects*. Duke University Press.

Agrawal, A., & Gibson, C. C. (1999). Enchantment and disenchantment: The role of community in natural resource conservation. *World Development*, 27(4), 629-649.

Arizona, Y., Wicaksono, M. T., & Vel, J. (2019). The role of indigeneity NGOs in the legal recognition of adat communities and customary forests in Indonesia. *Asia Pacific Journal of Anthropology*, 20(5), 487–506.

BBC News Indonesia. (2024). *Punan Batu: Penjaga Hutan Terakhir Kalimantan* [Video]. YouTube.

Borras Jr., S. M. (2006). The Philippine land reform in comparative perspective: Some conceptual and methodological implications. *Journal of Agrarian Change*, 6(1), 69-101.

Borras Jr., S. M., & Franco, J. C. (2005). Struggles for land and livelihood: Redistributive reform in agribusiness plantations in the Philippines. *Critical Asian Studies*, 37(3), 331-361.

Cali Tzay, J. F. (2024). Report of the Special Rapporteur on the rights of indigenous peoples, José Francisco Cali Tzay: Mobile Indigenous Peoples (A/79/160). United Nations General Assembly. https://undocs.org/A/79/160

CNN Indonesia. (2023). *Punan Batu, Komunitas Adat Nomaden Terakhir di Kalimantan* [Video]. YouTube.

Dinsos Kaltara. (2025). *Suku Punan Batu Benau Kalimantan Utara.* [Video]. YouTube.

de Certeau, M. (1984). *The practice of everyday life*. University of California Press.

Dove, M. R. (2006). Indigenous people and environmental politics. *Annual Review of Anthropology*, 35, 191-208.

Ferguson, J. (1996). *The anti-politics machine: "Development," depoliticization, and bureaucratic power in Lesotho*. University of Minnesota Press.

Fisher, M. R. (2019). *Beyond recognition: Indigenous land rights and changing landscapes in Indonesia* [Doctoral dissertation, University of Hawai'i at Manoa].

Fisher, M. R., & van der Muur, W. (2020). Misleading icons of communal lands in Indonesia: Implications of adat forest recognition from a model site in Kajang, Sulawesi. *Asia Pacific Journal of Anthropology*, 21(1), 55–76.

Foucault, M. (1991). Governmentality. In G. Burchell, C. Gordon, & P. Miller (Eds.), *The Foucault effect: Studies in governmentality* (pp. 87-104). University of Chicago Press.

Fraser, N. (2000). Rethinking recognition. *New Left Review*, 3, 107-120.

Gaventa, J. (2006). Finding the spaces for change: A power analysis. *IDS Bulletin*, 37(6), 23-33.

Hall, D., Hirsch, P., & Li, T. M. (2011). *Powers of exclusion: Land dilemmas in Southeast Asia*. NUS Press.

Ingold, T. (2000). *The perception of the environment: Essays on livelihood, dwelling and skill*. Routledge.

Keck, M. E., & Sikkink, K. (1998). *Activists beyond borders: Advocacy networks in international politics*. Cornell University Press.

Kompas TV. (2023). *Pengakuan Masyarakat Adat Punan Batu* [Video]. YouTube.

Kusuma, P., Cox, M. P., Barker, G., Sudoyo, H., Lansing, J. S., & Jacobs, G. S. (2023). Deep ancestry of Bornean hunter-gatherers supports long-term local ancestry dynamics. *Cell Reports*, 42(11), 113346.

Lansing, J. S., Jacobs, G. S., Downey, S. S., Norquest, P. K., Cox, M. P., Kuhn, S. L., ... & Kusuma, P. (2022). Deep ancestry of collapsing networks of nomadic hunter–gatherers in Borneo. *Evolutionary Human Sciences, 4*, e9. doi:10.1017/ehs. 2022.3

Li, T. M. (2007). *The will to improve: Governmentality, development, and the practice of politics*. Duke University Press.

Li, T. M. (2014). Anthropological engagements with development. *Anthropologie & développement*, (37-38-39), 227-240.

Li, T. M. (2020). The price of un/freedom: Indonesia's colonial and contemporary plantation labor regimes. *Comparative Studies in Society and History*, 62(2), 245-276.

Maryudi, A., & Fisher, M. R. (2020). The power in the interview: A practical guide for identifying the critical role of actor interests in environment research. *Forest and Society*, 4(1), 142–150.

Mosse, D. (2004). Is good policy unimplementable? Reflections on the ethnography of aid policy and practice. *Development and Change*, 35(4), 639-671.

NeT TV. (2024). *Punan Batu Meraih Kalpataru 2024* [Video]. YouTube.

Povinelli, E. A. (2002). *The cunning of recognition: Indigenous alterities and the making of Australian multiculturalism*. Duke University Press.

Ring, M. S., Hendartono, K., Om, M. A., Aries, M., Sjamsuri, A., Parti, A., ... & Berau Daerah tingkat Kalimantan Timur, K. I. (1964). *Laporan hasil survey terhadap suku terasing Punan*. Government Report.

Riyanto, G. (2025). Indigeneity as a sphere of differences: State enclosure and counter-enclosure of rural spaces in Indonesia. *Anthropological Theory*, 14634996251313825.

Sahide, Muhammad Alif K., Fisher, M. R., Verheijen, B., Maryudi, A., Kim, Y.-S., & Wong, G. Y. (2020a). Sequential power analysis framework in assessing social forestry outcomes. *MethodsX*, *7*(100917), 100917. https://doi.org/10.1016/j.mex.2020.100917

Sahide, M. A. K., Fisher, M. R., Erbaugh, J. T., Intarini, D., Dharmiasih, W., Makmur, M., ... & Maryudi, A. (2020c). The boom of social forestry policy and the bust of social forests in Indonesia: Developing and applying an access-exclusion framework to assess policy outcomes. *Forest Policy and Economics*, 120, 102290.

Sahide, M. A. K., Fisher, M. R., Supratman, S., Yusran, Y., Pratama, A. A., Maryudi, A., ... & Kim, Y. S. (2020b). Prophets and profits in Indonesia's social forestry partnership schemes: Introducing a sequential power analysis. *Forest Policy and Economics*, 115, 102160.

Scott, J. C. (1985). *Weapons of the weak: Everyday forms of peasant resistance*. Yale University Press.

Scott, J. C. (1998). *Seeing like a state: How certain schemes to improve the human condition have failed*. Yale University Press.

Sellato, B. (2001). *Forest, resources and people in Bulungan: Elements for the history of settlement, trade and social dynamics in Borneo, 1880-2000*. CIFOR.

Sylvain, R. (2014). Essentialism and the indigenous politics of recognition in Southern Africa. *American Anthropologist*, *116*(2), 251-264.

Tsing, A. L. (2005). *Friction: An ethnography of global connection*. Princeton University Press.

YKAN. (2022). *Punan Batu: The Last Nomads of Borneo* [Documentary]. YouTube.

Click here to access/download
Co-submission to MethodsX
MethodsX Article 2.docx

Declaration of interests

☒The authors declare that they have no known competing financial interests or personal relationships that could have appeared to influence the work reported in this paper.

☐The authors declare the following financial interests/personal relationships which may be considered as potential competing interests:
